# Classification of Estrogen Receptor-Positive Breast Cancer Based on Immunogenomic Profiling and Validation at Single-Cell Resolution

**DOI:** 10.3389/fcell.2021.722841

**Published:** 2021-09-21

**Authors:** Xianxiong Ma, Hengyu Chen, Ming Yang, Zunxiang Ke, Mengyi Wang, Tao Huang, Lei Li

**Affiliations:** ^1^Department of Gastrointestinal Surgery, Union Hospital, Tongji Medical College, Huazhong University of Science and Technology, Wuhan, China; ^2^Department of Breast and Thyroid Surgery, The Second Affiliated Hospital of Hainan Medical University, Haikou, China; ^3^Department of Pancreatic Surgery, Union Hospital, Tongji Medical College, Huazhong University of Science and Technology, Wuhan, China; ^4^Department of Emergency Surgery, Union Hospital, Tongji Medical College, Huazhong University of Science and Technology, Wuhan, China; ^5^Department of Breast and Thyroid Surgery, Union Hospital, Tongji Medical College, Huazhong University of Science and Technology, Wuhan, China

**Keywords:** breast cancer, immune signature, TCGA, GEO, molecular subtypes, ER+, genomic mutation, single-cell sequencing

## Abstract

**Background:** The aim of this paper was to identify an immunotherapy-sensitive subtype for estrogen receptor-positive breast cancer (ER+ BC) patients by exploring the relationship between cancer genetic programs and antitumor immunity via multidimensional genome-scale analyses.

**Methods:** Multidimensional ER+ BC high-throughput data (raw count data) including gene expression profiles, copy number variation (CNV) data, single-nucleotide polymorphism mutation data, and relevant clinical information were downloaded from The Cancer Genome Atlas to explore an immune subtype sensitive to immunotherapy using the Consensus Cluster Plus algorithm based on multidimensional genome-scale analyses. One ArrayExpress dataset and eight Gene Expression Omnibus (GEO) datasets (GEO-meta dataset) as well as the Molecular Taxonomy of Breast Cancer International Consortium dataset were used as validation sets to confirm the findings regarding the immune profiles, mutational features, and survival outcomes of the three identified immune subtypes. Moreover, the development trajectory of ER+ BC patients from the single-cell resolution level was also explored.

**Results:** Through comprehensive bioinformatics analysis, three immune subtypes of ER+ BC (C1, C2, and C3, designated the immune suppressive, activation, and neutral subtypes, respectively) were identified. C2 was associated with up-regulated immune cell signatures and immune checkpoint genes. Additionally, five tumor-related pathways (transforming growth factor, epithelial–mesenchymal transition, extracellular matrix, interferon-γ, and WNT signaling) tended to be more activated in C2 than in C1 and C3. Moreover, C2 was associated with a lower tumor mutation burden, a decreased neoantigen load, and fewer CNVs. Drug sensitivity analysis further showed that C2 may be more sensitive to immunosuppressive agents.

**Conclusion:** C2 (the immune activation subtype) may be sensitive to immunotherapy, which provides new insights into effective treatment approaches for ER+ BC.

## Introduction

Breast cancer (BC) is the most common cause of cancer-related death among females worldwide (Torre et al., 2017). The 5-year survival rate of BC patients is poor according to 2019 data (DeSantis et al., 2019). Estrogen receptor-positive (ER+) BC is the most common subtype of BC, accounting for approximately 75% of all BC cases (Burstein et al., 2010). The current treatments for ER+ BC include surgery, chemotherapy, and molecular targeted therapy (Bayraktar et al., 2019). However, treatment has been hindered by resistance in ER+ BC, which is related to the molecular heterogeneity and complex biological processes in these cases (Koren and Bentires-Alj, 2015). Thus, novel treatments are needed to improve the prognosis of ER+ BC patients.

Endocrine treatment is an important targeted therapy for patients with ER+ BC. However, quite a few BC patients with localized disease and most BC patients with metastasis develop resistance to endocrine therapy (No authors listed, 1998; Hurvitz and Pietras, 2008; Osborne and Schiff, 2011). Immunotherapy has gradually attracted the interest of many BC researchers. It is well known that an active immune microenvironment can hinder tumor growth and metastasis. Studies have suggested that tumor-infiltrating lymphocytes (TILs) were correlated with better cancer prognosis (Fridman et al., 2012). For example, Chu et al. (2019) reported that CD103(+) TILs predicted favorable prognosis in patients with esophageal squamous cell carcinoma. Hida et al. (2019) suggested that diffusely distributed TILs were a marker of improved prognosis in triple-negative BC (Adams et al., 2015). Loi et al. (2013) conducted a phase III randomized adjuvant trial of lymph node-positive BC patients (comparing doxorubicin plus docetaxel- vs doxorubicin-based chemotherapies) and revealed the prognostic and predictive values of TILs; the ER+ BC patients generally had low TIL levels, though a small proportion had very high TIL levels. However, no studies have reported a clear association between TILs and prognosis in ER+ BC patients’ prognoses thus far, since the outcomes of the ER+ subgroup are highly heterogeneous (Ali et al., 2014; Dieci et al., 2015; Luen et al., 2016).

Therefore, our aim was to explore the relationship between the genomic landscape and antitumor immunity via multidimensional genome-scale analyses to identify an immune subtype of ER+ BC patients that may be sensitive to immunotherapy. RNA-sequencing data from The Cancer Genome Atlas (TCGA) database was used to identify three discrete immune subtypes of ER+ BC (regarded as immune suppressive, activation, and neutral phenotypes). Somatic mutation data and copy number variation (CNV) data were used to explore the associations between genetic features and the three immune subtypes. Additionally, a Gene Expression Omnibus (GEO)-meta dataset (composed of nine small datasets) and a Molecular Taxonomy of BC International Consortium (METABRIC) dataset were used as validation sets to confirm the value of the three identified immune subtypes. Moreover, the drug sensitivity of each ER+ BC immune subtype and the development trajectory of ER+ tumor microenvironment were investigated. The results improve our understanding of the immune microenvironment of the primary tumors in ER+ BC patients and provide new insights into immunotherapy for ER+ BC.

## Materials and Methods

### Data Presentation and Filtering

#### TCGA Dataset

Level 3 multidimensional BC high-throughput data including gene expression profiles, CNV data, single-nucleotide polymorphism (SNP) mutation data, and relevant clinical information were downloaded from TCGA using the “TCGAbiolinks” package (Colaprico et al., 2016). We excluded samples based on the following criteria: (1) incomplete overall survival (OS) or recurrence-free survival (RFS) data; (2) no ER+ BC samples; (3) para-cancer tissue samples of BC patients; and (4) datasets containing less than 40 samples. Thereafter, immune-associated genes were retrieved from the ImmPort database^1^ (Bhattacharya et al., 2014). Eventually, gene expression data involving 1,811 immune-associated genes and 787 BC patients were used as the training dataset to identify a potentially immunotherapy-sensitive immune subtype among ER+ BC patients.

#### GEO-Meta Dataset

An ArrayExpress dataset (E-TABM-158, 83 samples) and eight GEO datasets (GSE1456, GSE2034, GSE2603, GSE45255, GSE4922, GSE6532, GSE7390, and GSE9195, containing 77, 209, 46, 58, 211, 201, 134, and 77 samples, respectively) along with a METABRIC dataset (1,435 samples) were used as external validation sets to confirm the findings regarding the immune profiles, mutational features, and survival outcomes of the identified immune subtypes. As both GEO and ArrayExpress data were obtained using an Affymetrix microarray chip, we integrated the data to create the GEO-meta dataset (1,096 samples in total) for subsequent analyses. All GEO-meta sub-datasets contained progression-free survival (PFS)/RFS/disease-free survival (DFS) information, whereas only a few GEO-meta sub-datasets contained OS data. Therefore, only PFS/RFS/DFS were used to conduct a survival analysis in the GEO-meta dataset (while OS data were also used in the corresponding TCGA and METABRIC analyses). The GEO-meta ER+ BC samples with complete PFS/RFS/DFS data were included in the following analysis.

#### METABRIC Dataset

Level 3 METABRIC data including gene expression profiles, CNV data, SNP mutation data, and matched clinical information were obtained from the cBioPortal database (Pereira et al., 2016). The eligible criteria were the same as above. The detailed clinical information of all the included samples is shown in Supplementary Table 1.

### Data Preprocessing

The immune-associated genes from the ImmPort database were used to identify the immune subtypes of ER+ BC and the immune cell profiles of these subtypes. The raw count matrix of level 3 TCGA mRNA data was converted to transcripts per million data. Genes with no expression in >50% of the samples were removed. Consequently, a TCGA training dataset consists of 1,295 immune-associated genes, and 787 ER+ BC samples were used in the cluster analysis. Regarding the GEO-meta sub-datasets, the raw data (CEL files) were normalized using the “rma” function in the “affy” package (Gautier et al., 2004). Regarding the METABRIC dataset, the normalized gene expression data were manually obtained directly from the cBioPortal website and used for subsequent analysis. In addition, the batch effects between different datasets were eliminated with the ComBat empirical Bayes method using the “sva” package (Chakraborty et al., 2012).

### Identification of ER+ BC Subtypes in the Training Dataset and Validation of Molecular Subtypes in the GEO-Meta Dataset and METABRIC Dataset

To identify ER+ BC subtypes based on immune-associated gene expression profiles, the “ConsensusClusterPlus” 1.50.0 package (Wilkerson and Hayes, 2010) was applied to generate a consensus matrix plot. This involved 1,000 iterations, a maximum of eight clusters, sampling of 80% of the samples at each iteration, and use of the partitioning around medoids clustering method. The optimal cluster number was determined based on cumulative distribution function curves of the consensus score. The samples from two validation datasets were then classified according to specified classifiers trained in the TCGA dataset by using diagonal linear discriminant analysis (DLDA), which is a machine learning approach available in the “classpredict” package developed using BRB-ArrayTools modules (Simon et al., 2007).

### Immune Profiles in ER+ BC Subtypes

First, the “GSVA” package and single-sample gene set enrichment analysis (ssGSEA; Bindea et al., 2013) were conducted with the expression signatures of 24 types of immune cells from a previous study (Hanzelmann et al., 2013). The resultant enrichment score for each of the 24 immune cell signatures represented the absolute enrichment of a particular gene set in each sample in the datasets (Subramanian et al., 2005). Second, for further validation, the “MCPcounter” package was also used to evaluate the absolute abundance of eight immune cell populations and two stromal cell populations based on gene expression profiles (Becht et al., 2016). Third, the “ESTIMATE” package was used to the estimate immune score and stromal score (Yoshihara et al., 2013), which were compared between subtypes. Fourth, the expression level of about 30 potentially targetable immune checkpoint molecules was compared between each subtype (Kim et al., 2019; Toor et al., 2019). Lastly, tumor mutation burden (TMB), neoantigen load, and CNV were compared between the subtypes, with the predicted neoantigen loads being calculated based on a previous analysis (Rooney et al., 2015). Thus, immune signature scores, immune checkpoint gene expression, and mutational features were compared between the three subtypes. The differences were assessed using the Mann–Whitney *U* test, with the Bonferroni correction being used to correct for multiple comparisons. Meanwhile, the LASSO regression model was used to construct a scoring system to quantify immune subtypes for individuals. The LASSO regression model was implemented via a publicly available R package “glmnet” (Engebretsen and Bohlin, 2019).

### Prediction of the Benefit of Each Subclass From Immunotherapy and Targeted Therapy

Data from 47 melanoma patients treated with immunotherapy (a programmed cell death protein [PD]-1 inhibitor or a cytotoxic T-lymphocyte-associated protein [CTLA]-4 inhibitor) were used to assess the potential responses to immunotherapy of our three immune subtypes. This was done by assessing the similarities in gene expression profiles between our subtypes and the melanoma patients using a SubMap analysis in GenePattern (Roh et al., 2017). Additionally, sensitivity to two ER+ BC-targeted drugs (tamoxifen and fulvestrant) was evaluated in a SubMap analysis based on Genomics of Drug Sensitivity in Cancer cell line data (Iorio et al., 2016). 48 BC cell lines were ranked based on the half maximal inhibitory concentration (IC50) value, with 50% of cell lines with the highest IC50 values being considered drug resistant and the remainder being considered drug sensitive.

### Single-Cell Development Analysis

We used single-cell data (GSE114725) to explore the development trajectory of the tumor microenvironment in ER+ BC patients. In total, 14,800 cells from four ER+ patients out of eight BC patients in the original dataset were included in our study. The raw count matrix of single-cell data was downloaded from the GEO database. We successfully annotated 3,903 cells using “SingleR” packages (Aran et al., 2019), and a development trajectory analysis was performed using “Monocle” packages (Qiu et al., 2017). Furthermore, the annotation results regarding the 3,903 cells reported by Azizi et al. (2018) were used to validate our annotation results.

### Additional Statistical Analyses

Chi-square and Fisher’s exact tests were used to assess the associations between conventional clinical variables [age, ethnicity, cancer site, cancer stage, human epidermal growth factor receptor 2 (HER2) status, progesterone receptor status, and number of positive lymph nodes] and our three immune subtypes. Kaplan–Meier curves and log-rank tests were used to compare the survival of patients with the three immune subtypes. All analyses were performed using R software, unless otherwise specified.

## Results

### ER+ BC Were Divided Into Three Subtypes Based on the ConsensusClusterPlus Algorithm

**FIGURE 1:**
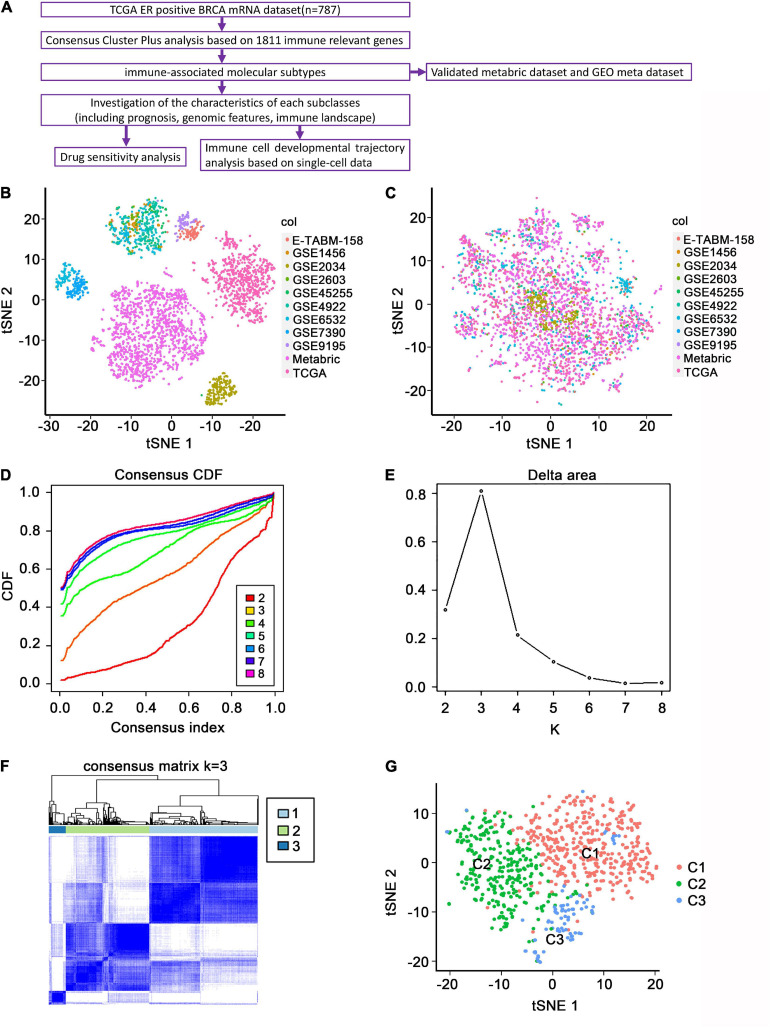
Identification of immune-associated subtypes of ER+ BC in the TCGA dataset. **(A)** Flowchart of the present study. **(B)** The t-SNE distribution of all the enrolled patients before removing batch effects. Each point represents a single sample; different colors represent the included sub-datasets. **(C)** The t-SNE distribution of all the enrolled patients after removing batch effects. **(D)** The cumulative distribution function (CDF) curves in consensus cluster analysis. CDF curves of consensus scores by different subtype numbers (*k* = 2, 3, 4, 5, 6, 7, and 8) were displayed. **(E)** Relative change in area under the CDF curve for *k* = 2–8. **(F)** The consensus score matrix of all samples when *k* = 3. The higher the consensus score was, the more likely they were assigned to the same group. **(G)** The t-SNE distribution of TCGA ER+ BC samples by expression profile of global immune genes. Each point represents a single sample; different colors represent the three subtypes.

The study flowchart is presented in Figure 1A. The batch effect was removed from the standardized data before consensus clustering. The t-distributed stochastic neighbor embedding (t-SNE) performance before and after removing the batch effect is shown in Figures 1B,C, respectively. The expression profiles of 1,295 immune-associated genes were used to classify patients into *K* that consists of two to eight clusters, using the ConsensusClusterPlus algorithm (Figure 1D). *K* = 3 was optimal based on the cumulative distribution function curves of the consensus score (Figures 1E,F). The three subtypes were presented on a two-dimensional scatter plot based on the t-SNE algorithm and could be distinguished well (Figure 1G), which was largely concordant with the previous consensus clustering shown in Figures 1E,F.

The distributions of conventional clinic-related variables among the three molecular subtypes from the TCGA cohort are shown in Table 1. The association between age and three subtypes was significant (*p* = 0.0003). Meanwhile, there were also significant differences for age, HER2 status, number of positive lymph nodes, and ethnicity in C2 patients (*p* = 0.0003, *p* < 0.001, and *p* < 0.0015, respectively), whereas the other clinical variables are statistically non-significant.

**TABLE 1:** Association of three immune subtypes with clinical variables in the TCGA and METABRIC cohorts.

Characteristic		TCGA cohort				METABRIC cohort			
		C1	C2	C3	*p*-value	C1	C2	C3	*p*-value
Age					0.0003				<0.001
	≥60	222	120	48		595	250	45	
	<60	184	177	36		303	221	21	
Site					0.41				0.31
	Left	216	143	44		459	220	33	
	Right	190	154	40		390	227	32	
	Unknown					49	24	1	
Stage					0.69				0.96
	Stage I	74	56	10		229	135	18	
	Stage II	229	160	49		372	207	26	
	Stage III	88	71	24		44	23	1	
	Stage IV	6	4	1		6	3	0	
	Unknown	9	6	0		1	1	0	
HER2 status					0.0003				0.3
	Positive	76	31	13		58	38	7	
	Negative	202	186	35		840	433	59	
	Indeterminate	128	80	36					
PR status					0.32				0.26
	Positive	342	259	66		621	307	42	
	Negative	62	37	18		277	164	24	
	Indeterminate	2	1	0					
No. of positive lymph nodes					<0.001				0.12
	0	149	127	30		491	250	44	
	≥1	170	146	46		407	221	22	
	Unknown	87	24	8					
Ethnicity					0.0015				
	Hispanic/Latino	15	12	3					
	Not Hispanic/Latino	301	237	78					
	Unknown	90	48	3					

*HER2: human epidermal growth factor receptor 2; PR: progesterone receptor.*

### Immune Cell Profiles of the Three Subtypes

**FIGURE 2:**
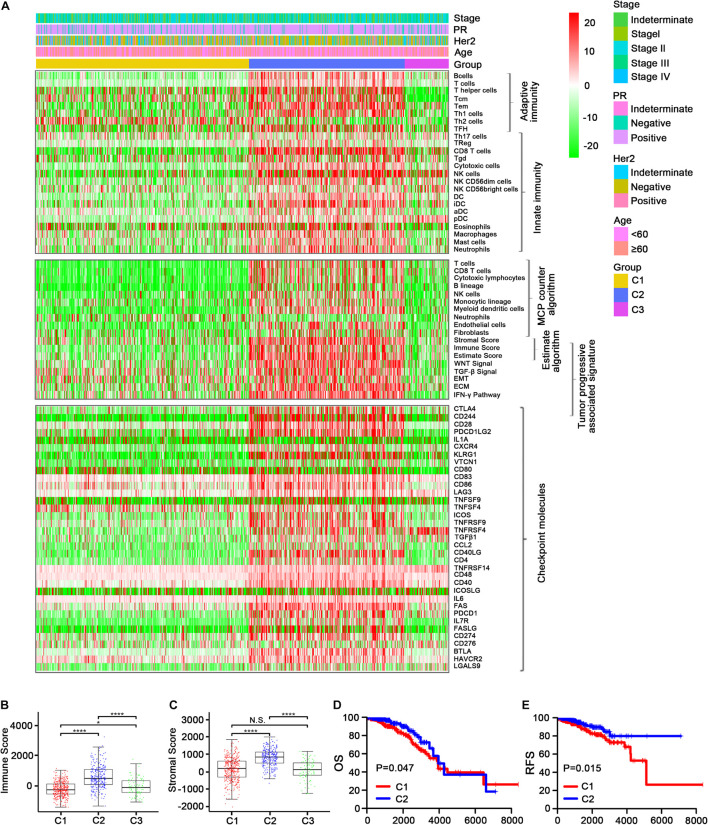
Immune profiles of the three identified subclasses in the TCGA dataset. **(A)** The enrichment scores of 24 immune cell signatures and clinicopathologic features across three subclasses were presented in the upper panel. The middle panel indicated the abundance profile of 10 immune-related cells (eight immune cells and two stromal cells, MCPcounter algorithm) and immune-associated scores (ESTIMATE algorithm) as well as enrichment score of tumor progression-associated pathways (ssGSEA algorithm). The lower panel displayed the expression profile of immune checkpoint molecules across three subclasses. The heatmap represents the relative value of indicators, with red for high value and green for low value. Boxplot of the immune score **(B)** and stromal score **(C)** from ESTIMATE of three subclasses. The horizontal lines indicated 5, 25, 50, 75, and 95%. Comparisons between subtypes were performed by the Kruskal–Wallis test, and the *p*-values were labeled above each boxplot with asterisks (N.S. represents no significance, *p < 0.05, and ****p < 0.0001). Kaplan–Meier curves show the distinct OS **(D)** and RFS **(E)** of patients in the immune activation (C2) class and immune suppressive class (C1). *p*-values were obtained using the log-rank test.

The enrichment scores regarding the 24 immune cell signatures for each TCGA sample are shown in Figure 2A (top). Most of these immune signatures were up-regulated in C2 compared with C1 and C3, while Th2 cells were the most highly up-regulated cells in C1 and plasmacytoid dendritic cells (pDCs) and CD56 (bright) natural killer (NK) cells were mainly up-regulated cells in C3 (Supplementary Figure 1). Additionally, the distribution of eight immune cells and two stromal cells identified using the MCPcounter algorithm confirmed our initial immune cell signature results (Figure 2A, middle, and Supplementary Figure 2). Thereafter, the ESTIMATE algorithm was used to calculate immune and stromal scores. As expected, C2 had a higher score than C1 and C3 (*p* < 0.001; Figures 2A–C).

We also examined the expression level of >30 immune checkpoint molecules that have been reported to play important roles in T-cell regulation in the TCGA dataset (Kim et al., 2019; Toor et al., 2019). Most of the immune checkpoint molecules were more obviously up-regulated in C2 than in C1, and TNFRSF14 was the most regulated immune checkpoint molecule in C3 (Figure 2A, bottom; Supplementary Figures 3A, 4, 5). Taking into account the consistency of the up-regulated immune cell signatures and the immune checkpoint molecules in C2, followed by C3 and then C1, we designated C1, C2, and C3 as the immune suppressive, activation, and neutral subtypes, respectively.

We then compared the survival data between the three immune subtypes. Unexpectedly, both OS and DFS were longer for C2 (the immune activation subtype) than for C1 (the immune suppressive subtype; Figures 2D,E and Supplementary Figures 3B,C). The findings were contrary to current mainstream perception (Zhang et al., 2020), which suggests that increased immune cell infiltration leads to worse prognosis. Meanwhile, the samples were converted into dichotomous variables (C2 group and non-C2 group), and an eight-gene immune signature was developed and used to construct a scoring system to quantify immune subtypes for individuals using the following risk score formula: 0.10394^∗^GAST + 0.56421^∗^HFE + 0.80564^∗^PLXNA3 + 0.40455^∗^P LXNC1 + 0.1667^∗^SEMA3A + 0.26431^∗^TNFRSF11B + 1.16759^∗^Z C3HAV1 − 0.12962^∗^CCL14 (Supplementary Figures 3D,E).

Studies have reported that tumor-associated signatures such as the transforming growth factor (TGF)-β signature and epithelial–mesenchymal transition (EMT) signature play important roles in tumor progression and drug resistance (Oshimori et al., 2015; Xu et al., 2018; Tripathi et al., 2019). Unsurprisingly, the five tumor progression pathways [TGF-β signaling, EMT, extracellular matrix (ECM), interferon (IFN)-γ, and WNT signaling] were more activated in C2 than in C1 and C3 (Figure 2A, middle, and Supplementary Figure 2). This indicates that C2 may involve relatively advanced tumor stages, leading to increased immune cell recruitment to combat the tumor cells.

### C2 Was Associated With a Lower TMB, a Decreased Neoantigen Load, and Fewer CNVs

**FIGURE 3:**
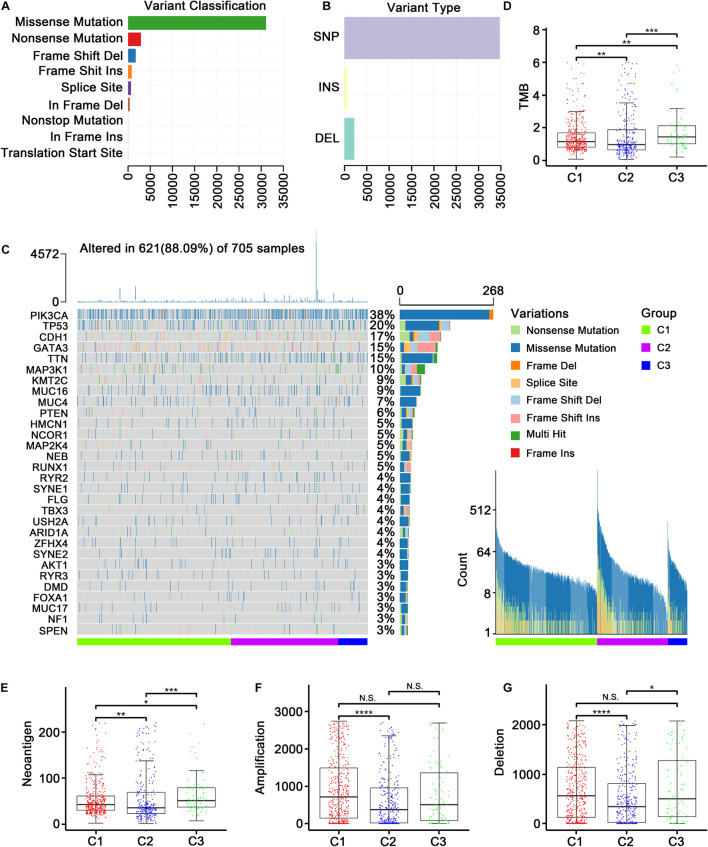
Genomic and mutational landscape of three subtypes in TCGA cohort. **(A)** Barplot of variant classification distribution among TCGA ER+ BC patients. **(B)** Bar plot of variant type distribution among TCGA ER+ BC patients. **(C)** Oncoprint of mutation status of top 30 highly mutated genes across three subtypes (left) and ordered by mutation load (right). **(D–G)** Represent the boxplot of tumor mutation load, neoantigen load, copy number amplifications and copy number deletions, respectively. Comparisons between subtypes were performed by the Kruskal–Wallis test, and the *p*-values were labeled above each boxplot with asterisks (N.S. represents no significance, *p < 0.05, **p < 0.01, ***p < 0.001, and ****p < 0.0001).

Studies have reported that the genomic and mutational landscape is related to antitumor immunity. For example, Takahashi et al. (2020) revealed an association between a biologically aggressive phenotype in BC with a high mutation rate and an anticancer immunity counterbalance. Additionally, aneuploidy has been reported to be involved in immune evasion, which may reduce the effect of immunotherapy (Davoli et al., 2017). To explore whether the TMB, neoantigen load, and CNV affect the immune microenvironment in ER+ BC patients in the TCGA dataset, we conducted a comprehensive analysis to examine the associations between these factors and the immune subtypes. The variants were mostly missense mutations, and SNPs were the most common variant type (Figures 3A,B). Figure 3C presented a panoramic view of ER+ mutations in each immune subtype: PIK3CA, TP53, CDH1, GATA3, and TTN were the genes with the highest mutation rates. C2 had a significantly lower TMB (Figure 3D) and a significantly decreased neoantigen load (Figure 3E) compared with C1. Furthermore, C2 had the significantly lowest CNV regarding both amplification and deletion (Figures 3F,G).

We further applied the MutSigCV algorithm to identify driver genes based on the TCGA mutation data, and 27 mutated genes were identified (FDR < 0.05, Supplementary Table 2). Among these genes, two (CDH1 and PIK3R1) were the marker genes (Supplementary Table 3) of three identified immune subtypes. It should be noted that CDH1 was a marker gene in all three subtypes. In other words, CDH1 expression was significantly different in each of the three subtypes, suggesting that CDH1 may be the mutation driver gene that modified the three immune subtypes. Unlike CDH1, PIK3R1 was a specific marker gene of the immune neutral subtype.

### Three Immune Subtypes Were Validated in the GEO-Meta and METABRIC Datasets

The three immune subtypes identified in the TCGA training cohort were validated in two external cohorts using a DLDA classifier.

**FIGURE 4:**
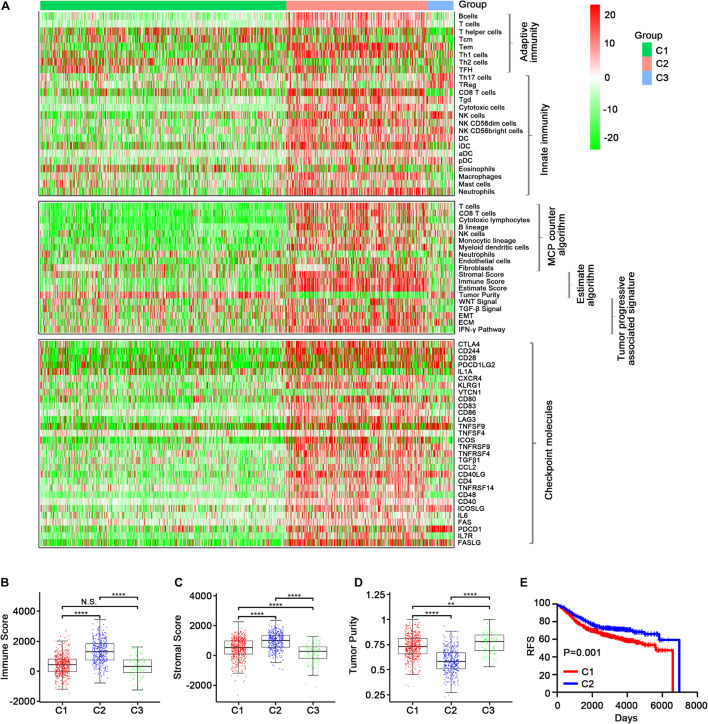
Validation of the three immune-related subtypes in the GEO-meta cohort. **(A)** The enrichment scores of 24 immune cell signatures across three subclasses were presented in the upper panel. The middle panel indicated the abundance profile of 10 immune-related cells (eight immune cells and two stromal cells, MCPcounter algorithm) and immune-associated scores (ESTIMATE algorithm) as well as enrichment score of tumor progression-associated pathways (ssGSEA algorithm). The lower panel displayed the expression profile of immune checkpoint molecules across three subclasses. The heatmap represents the relative value of indicators, with red for high value and green for low value. Boxplot of the immune score **(B)** and stromal score **(C)** as well as tumor purity **(D)** from ESTIMATE algorithm of three subclasses. **(E)** Kaplan–Meier curves show the distinct RFS of patients in the immune activation (C2) class and immune suppressive class (C1). *p*-values were obtained using the log-rank test. (N.S. represents no significance, values are significant at **P < 0.01, and ****p < 0.0001 as indicated).

In the GEO-meta cohort, consistent with the TCGA cohort, C2 was shown to have high levels of most of the immune cell signatures (Figure 4A, top), while Th2 cells were most distinctly up-regulated in C1 and CD56 (bright) NK cells were most obviously up-regulated in C3. Additionally, Th17, Treg, and NK cells were also up-regulated in C3. Eosinophils were the most highly up-regulated cells in C1 (Supplementary Figure 6). Similar results were obtained using the MCPcounter algorithm except that the neutrophils were down-regulated in C2 (Figure 4A, middle). The ESTIMATE algorithm also demonstrated that the immune, stromal score and tumor purity were highest in C2 (Figure 4A, middle; Figures 4B–D, Supplementary Figure 7). Moreover, C2 had high expression levels of the immune checkpoint molecules (Figure 4A, bottom; Supplementary Figures 8A, 9, 10), and the Kaplan–Meier survival analysis revealed that C2 was associated with improved prognosis (Figure 4E and Supplementary Figure 8B).

**FIGURE 5:**
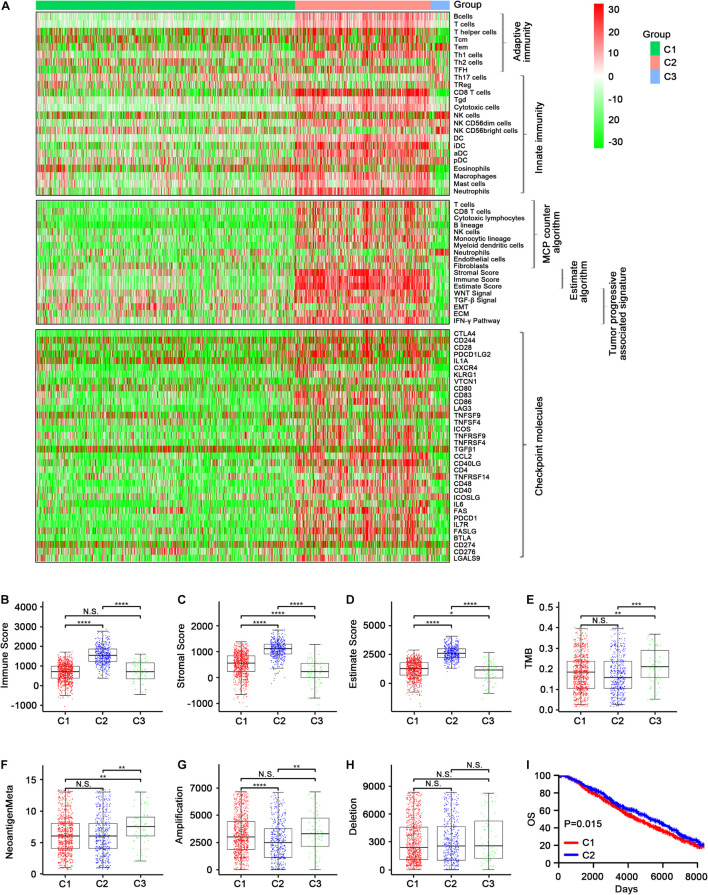
Validation of the three immune-related subtypes in the Metabric cohort. **(A)** The enrichment scores of 24 immune cell signatures across three subclasses were presented in the upper panel. The middle panel indicated the abundance profile of ten immune-related cells (eight immune cells and two stromal cells, MCPcounter algorithm) and immune-associated scores (ESTIMATE algorithm) as well as enrichment score of tumor progression-associated pathways (ssGSEA algorithm). The lower panel displayed the expression profile of immune checkpoint molecules across three subclasses. The heatmap represents the relative value of indicator, with red for high value and green for low value. **(B–D)** Showed boxplot of immune score and stromal score as well as estimate score from ESTIMATE algorithm of three subclasses, respectively. **(E–H)** Represent the boxplot of tumor mutation load, neoantigen load, copy number amplifications, and copy number deletions, respectively. Comparisons between subtypes were performed by the Kruskal–Wallis test, and the *p*-values were labeled above each boxplot with asterisks. **(I)** Kaplan–Meier curves show the distinct OS of patients in immune activation (C2) class and immune suppressive class (C1). *p*-values were obtained using the log-rank test. (N.S. represents no significance, *p < 0.05, **p < 0.01, ***p < 0.001, and ****p < 0.0001).

In the METABRIC cohort, a majority of immune cells, most immune checkpoint molecule expression levels, and the immune score were increased in C2 (Figures 5A–D and Supplementary Figures 11, 12, 14, 15). TMB and neoantigen load were significantly lower in C1 and C2 than in C3, while there was no significant difference in TMB between C1 and C2, although it was lower in C2 than in C1 (Figures 5E,F and Supplementary Figure 13). Regarding CNV, C2 had the lowest CNV regarding amplification, which was significant (Figure 5G), while no significant differences in deletion were observed among C1, C2, and C3 (Figure 5H). In addition, the OS was better in C2 than in C1 (Figure 5I and Supplementary Figure 13E). The distributions of conventional clinical variables among the three immune subtypes in the METABRIC cohort are shown in Table 1.

The observations in both GEO-meta and METABRIC datasets were highly consistent with those in the TCGA cohort, indicating the robustness of our immune subtype classification.

### Predictions of Differential Sensitivity to Immunotherapy and Targeted Therapies

**FIGURE 6:**
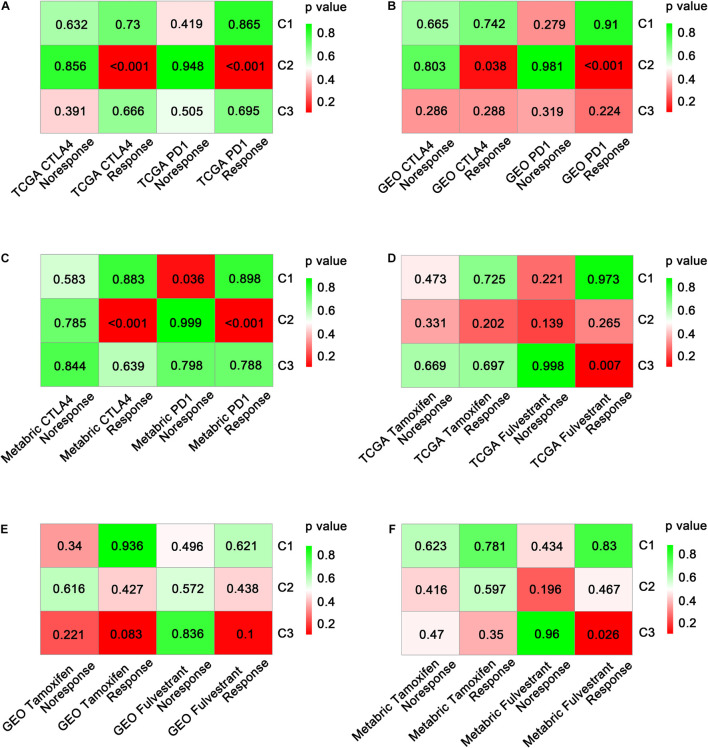
Prediction of the benefit of each subtype from immunotherapy and targeted therapy. Significance of each subclass’s drug sensitivity toward immune inhibitors (PD-1 and CTLA-4) in TCGA **(A)**, GEO **(B)**, and METABRIC **(C)** cohorts. Significance of each subclass’s drug sensitivity toward targeted drugs (tamoxifen and fulvestrant) in TCGA **(D)**, GEO **(E)**, and METABRIC **(F)** cohorts.

As the effectiveness of immunotherapy varies due to different immune cell infiltration patterns (Li J. et al., 2018) and expression levels of immune checkpoint molecules, it was important to explore the potential immunotherapy sensitivity of each immune subtype. Using SubMap analysis, we mapped the expression profiles of three immune subtypes (C1, C2, and C3) with another published cohort involving 47 melanoma patients who were treated with a PD-1 or CTLA-4 immune checkpoint inhibitor. A significant association was observed when comparing the expression profile of C2 patients with that of the CTLA-4 inhibitor-responsive group (*p* < 0.05) in all three datasets assessed (Figures 6A–C and Supplementary Table 4). This indicates that C2 patients are more likely to respond to anti-CTLA-4 immunotherapy. Similar results were obtained when comparing the expression profile of C2 patients with that of the PD-1 inhibitor-responsive group, indicating that C2 patients may also respond to anti-PD-1 immunotherapy.

Moreover, we investigated the sensitivity of immune subtypes to targeted drugs (tamoxifen and fulvestrant) using SubMap analysis. Unexpectedly, C3 rather than C2 exhibited a significant association with the fulvestrant-sensitive group in both the TCGA and METABRIC cohorts (*p* < 0.05; Figures 6D–F). A similar but non-significant trend was observed in the GEO-meta cohort (*p* = 0.1; Figure 6E). Regarding tamoxifen, no significant associations were observed between any of the immune subtypes and the tamoxifen-sensitive or tamoxifen-resistant groups (Supplementary Table 4).

### Single-Cell Development Trajectory Analysis

**FIGURE 7:**
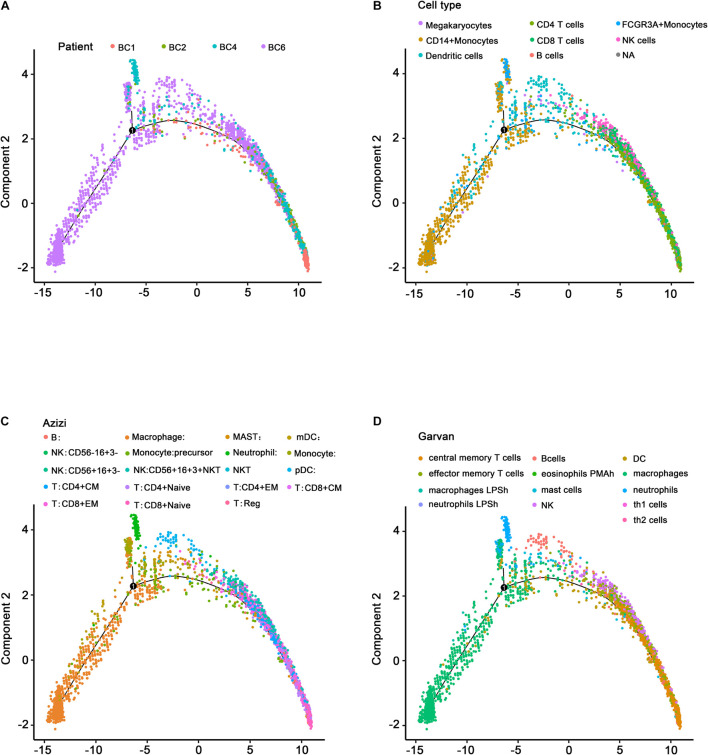
Single-cell development trajectory analysis of ER+ BC patients. **(A)** Development trajectory distribution of four ER+ BC patients. Different colors represent different patients. **(B)** Development trajectory annotated by the SingleR package. **(C)** Development trajectory annotated by Azizi et al. **(D)** Development trajectory annotated by Garvan. Different colors represent different immune microenvironment-associated cells.

The effectiveness of immunotherapy varies at different stages due to the different immune cell infiltration patterns and expression levels of immune checkpoint molecules (Li J. et al., 2018). The significant heterogeneity makes it difficult to identify effective treatment targets. However, single-cell data reflecting the diverse immune phenotypes help to avoid this issue. These data are useful for exploring tumor development trajectories and identifying more precise and effective treatment targets or biomarkers. The development trajectories of four patients, analyzed using Monocle, are presented in Figure 7A. As expected, the SingleR annotation results (Figure 7B) indicated similar development trajectories to those reported by Azizi et al. (Figure 7C) and Garvan et al. (Figure 7D and Supplementary Table 5), reflecting the same transition from innate immunity to adaptive immunity and the same immune cell infiltration pattern during tumor development. Three patients (BC1, BC2, and BC4) had most of the cells associated with adaptive immunity and were at a relatively late stage of the development trajectory, suggesting that these patients may be C2 patients (belonging to the immune activation subtype) or that these patients may have advanced-stage cancer and may be sensitive to immunotherapy.

## Discussion

In this study, we explored ER+ BC immune subtypes, identifying a subtype that may be sensitive to immunotherapy. Our ER+ BC subtype classification was developed based on level 3 multidimensional BC high-throughput data from the TCGA database. In addition, the GEO-meta dataset (composed of nine small datasets) and a METABRIC dataset were used to validate the identified immune subtypes. As a result, three subtypes of ER+ BC (C1, C2, and C3) were identified as immune suppressive, immune activation, and immune neutral subtypes, respectively, via a comprehensive bioinformatics analysis. Additionally, the immune cell signatures, activated signaling pathways, mutation features, and drug sensitivity of the subtypes were further explored. The results exhibited that C2 was related to many immune cell signatures and high expression levels of immune checkpoint genes, suggesting that C2 may be sensitive to CTLA-4 inhibitors and PD-1 inhibitors.

Previous studies have revealed several molecular subtypes of BC. For example, a study reported the genetic determinants of BC immune phenotypes based on integrative genome-scale analysis (Hendrickx et al., 2017), and Denkiewicz et al. (2019) identified BC subtype-specific microRNAs based on survival analysis to explore the roles of microRNAs in transcriptomic regulation. In our study, we mainly concentrated on the overall immune profiles, which may offer more detailed information about the immune landscape of ER+ BC. Among our three subtypes, C2 exhibited an overall up-regulated immune profile relative to C1 and C3, indicating that C2 has increased lymphocyte infiltration. Additionally, C2 was associated with lower TMB, a decreased neoantigen load, and fewer CNVs. These findings suggest that the tumor microenvironment of C2 exhibits an increased immune status. Moreover, Figure 2A shows that pDCs and CD56 (bright) NK cells were mainly up-regulated in C3 in the TCGA cohort. A previous study reported that the level of circulating CD56 (bright) NK cells was inversely correlated with survival in melanoma patients (de Jonge et al., 2019). Schuster et al. suggested that pDCs surrounded and infiltrated some tumors such as malignant melanoma, head and neck cancer, ovarian cancer, and BC. We speculated that subtype C3 ER+ BC patients may have an unfavorable prognosis, as the presence of pDCs has been reported to be associated with poor prognosis (under the premise that these cells are unstimulated; Schuster et al., 2019). Our results confirmed this speculation (Supplementary Figures 3C, 8B). In addition, Th2 cells were the most highly up-regulated cells in C1 rather than in C2. The relationship between Th2 cells and cancer prognosis differs among cancer types. For example, Schreck et al. (2009) found that increased Th2 cells were related to significantly increased DFS in classical Hodgkin’s lymphoma. In contrast, Chen et al. (2016) speculated that high expressions of Th2-related cytokines in hypopharyngeal cancer may contribute to cancer progression and metastasis, which may lead to poor prognosis. Those contradictory results may indicate that C2 was associated with a good prognosis, which indicated that the combined effect of immune cell infiltration in C2 may be favorable for prognosis.

A previous study indicated that neither neoantigen load nor TMB was related to T-cell response, while CNV may influence the immune response (Yang et al., 2020). Additionally, McGranahan et al. (2016) suggested that neoantigen quality instead of quantity could play a significant role in immune reactivity. In clinical practice, physicians may take immune molecular subtype, neoantigen quality, and CNV into consideration when identifying cancer patients, with a higher likelihood of responding to immunotherapy. Interestingly, identifying difference in the mutational features among immune subtypes may lead to biomarker identification. For example, Hao and Guo (2019) found that epidermal growth factor receptor mutation served as a novel prognostic factor related to immune infiltration in lower-grade glioma, while Zeng et al. (2016) suggested that the BRAF V600E mutation was associated with suppressive tumor immune microenvironment. We analyzed the TMB, neoantigen load, and CNV in the three immune subtypes. The result showed that C2 was significantly associated with a lower TMB, a decreased neoantigen load, and fewer CNVs in the TCGA and METABRIC datasets, which may provide insights into the identification of novel ER+ BC biomarkers.

Cancer immunotherapy aims to trigger a self-sustaining cancer-related immune response while minimizing therapy-associated autoinflammation (Karasaki et al., 2017). Several studies suggested that the immune checkpoints PD-1, CTLA-4, and T-cell immunoglobulin and mucin-domain containing-3 (TIM-3; Liu et al., 2017; Naoum et al., 2018; El Dika et al., 2019) are crucial indicators sustaining the pro-tumor immune microenvironment. They are also considered as perfect targets for carcinoma immunotherapy. Figure 6 indicates that, based on comparisons of expression profiles, the C2 patients are more likely to respond to anti-CTLA-4 and anti-PD-1 immunotherapy. However, the frequent resistance exhibited against immune checkpoint inhibitors indicates that PD-1 or CTLA-4-targeted monotherapy may not fully offset the immunosuppression in the tumor microenvironment (Li X. et al., 2018). We hypothesize that other related immune checkpoints could be targeted in order to increase antitumor immunity. Nevertheless, the identification of C2 (the immune activation subtype) in this study may help to guide the choice of monotherapy or combination therapy for ER+ BC patients. Further validation in clinical trials is required before clinical application.

Figure 2 showed that five tumor-related pathways (TGF-β signaling, EMT, ECM, IFN-γ, and WNT signaling) were more activated in C2 than in C1 and C3. Previous research suggested that the TGF-β pathway has bidirectional effects in cancers (Metelli et al., 2018). In premalignant cells, TGF-β served as a tumor suppressor by inhibiting cell proliferation and facilitating apoptosis, whereas, in advanced tumors, TGF-β facilitated metastasis and induced a protumor immune microenvironment (David and Massagué, 2018). Donato et al. (2020) suggested that proliferation and migration of neural crest-derived cells involved the activation of the EMT pathway. Schomberg et al. (2020) found that luteolin-mediated inhibition of melanoma cell growth may involve simultaneously affecting various pathways such as the ECM, oncogenic signaling, and immune response pathways. Gao et al. (2016) suggested that the loss of the IFN-γ pathway genes in tumor cells may underlie resistance to anti-CTLA-4 treatment. Sun et al. (2019) found that miR-22 and miR-214 targeting BCL9L inhibited proliferation, metastasis, and EMT by down-regulating Wnt signaling in colon cancer. Nevertheless, clinical evidence and clinical trials are required to verify the combined effects of the above five pathways on tumor immunotherapy. Further exploration of these pathways may help to develop targeted antitumor therapy.

This study has several advantages that need to be expounded upon. Firstly, this is the first study to comprehensively describe the immune profile of ER+ BC cases, with data from three databases (TCGA, GEO-meta, and METABRIC datasets) involving a very large sample (3,318 samples) being combined to identify the ER+ BC immune subtypes. In other words, we performed multiple validations involving multiple datasets in order to confirm the identified immune subtypes, which made our findings more reliable. Secondly, three algorithms (ssGSEA, MCPcounter, and ESTIMATE) were applied to investigate the immune cell signatures in each immune subtype, and similar results were obtained, which indicated the robustness of our immune subtype classification. Thirdly, we explored the characteristics of each subtype not only based on gene expression but also based on mutational features (TMB, neoantigen load, and CNV). Additionally, we predicted the drug susceptibility of each subtype and explored the developmental trajectory of BC patients. This multidimensional analysis provides a comprehensive picture of the clinical significance of each immune subtype and provides a foundation for improving clinical treatment.

## Conclusion

We identified and verified a novel immune subtype classification of ER+ BC, which involves three robust subtypes: the immune suppressive, activation, and neutral subtypes. Patients with C2 (the immune activation subtype) represent the optimal candidates for anti-PD-1 and anti-CTLA-4 immunotherapy. Our classification may help to predict the prognosis of ER+ BC patients and provide clinicians a new basis for making accurate clinical diagnoses and selecting optimal treatments such as immunotherapy.

## Data Availability Statement

The data that support the findings of this study are available in the TCGA, GEO, and METABRIC databases.

## Ethics Statement

This article does not contain any studies with human participants or animals performed by any of the authors.

## Author Contributions

XM and HC designed, extracted, analyzed, and interpreted the data from TCGA, GEO, and METABRIC databases. ZK and TH wrote the manuscript. MY and MW revised the manuscript. LL made substantial contributions to the conception of the work. XM, HC, and LL substantively revised it. All authors have read and approved the final manuscript.

## Conflict of Interest

The authors declare that the research was conducted in the absence of any commercial or financial relationships that could be construed as a potential conflict of interest. The reviewer WC declared a shared affiliation, with one of the authors, HC, to the handling editor at time of review.

## Publisher’s Note

All claims expressed in this article are solely those of the authors and do not necessarily represent those of their affiliated organizations, or those of the publisher, the editors and the reviewers. Any product that may be evaluated in this article, or claim that may be made by its manufacturer, is not guaranteed or endorsed by the publisher.

## Footnotes

1
http://www.immport.org

## Supplementary Material

Supplementary Figure 1 Boxplot of the distribution of 24 immune cells among three groups in the TCGA cohort (N.S. represents no significance, **p* < 0.05, ***p* < 0.01, ****p* < 0.001, and *****p* < 0.0001).

Supplementary Figure 2 Boxplot of the distribution of 10 immune-related cells and five tumor progression-related pathways among three groups in the TCGA cohort (N.S. represents no significance, **p* < 0.05, ***p* < 0.01, ****p* < 0.001, and *****p* < 0.0001).

Supplementary Figure 3 Heatmap of the top 10 marker genes of each subclass in the TCGA cohort **(A)** and Kaplan–Meier curves of each subclass **(B,C)**. Candidate immune gene selection using the LASSO regression model **(D,E)**.

Supplementary Figure 4 Boxplot of the distribution of 35 checkpoint molecules among three groups in the TCGA cohort (N.S. represents no significance, **p* < 0.05, ***p* < 0.01, ****p* < 0.001, and *****p* < 0.0001).

Supplementary Figure 5 Coloring single samples on a dimensional reduction plot according to 23 checkpoint molecules in the TCGA cohort. **(A)** The t-SNE distribution of TCGA ER+ BC samples by expression profile of global immune genes. Each point represents a single sample; different colors represent the three subclasses. **(B–X)** Coloring single samples on a dimensional reduction plot according to 23 checkpoint molecules.

Supplementary Figure 6 Boxplot of the distribution of 24 immune cells among three groups in the GEO-meta cohort (N.S. represents no significance, **p* < 0.05, ***p* < 0.01, ****p* < 0.001, and *****p* < 0.0001).

Supplementary Figure 7 Boxplot of the distribution of 10 immune-related cells and five tumor progression-related pathways among three groups in the GEO-meta cohort (N.S. represents no significance, **p* < 0.05, ***p* < 0.01, ****p* < 0.001, and *****p* < 0.0001).

Supplementary Figure 8 Heatmap of the top 10 marker genes of each subclass in the GEO-meta cohort **(A)** and Kaplan–Meier curves of each subclass **(B)**.

Supplementary Figure 9 Boxplot of the distribution of 30 checkpoint molecules among three groups in the GEO-meta cohort (N.S. represents no significance, **p* < 0.05, ***p* < 0.01, ****p* < 0.001, and *****p* < 0.0001).

Supplementary Figure 10 Coloring single samples on a dimensional reduction plot according to 23 checkpoint molecules in the GEO-meta cohort. **(A)** The t-SNE distribution of GEO-meta ER+ BC samples by expression profile of global immune genes. Each point represents a single sample; different colors represent the three subclasses. **(B–X)** Coloring single samples on a dimensional reduction plot according to 23 checkpoint molecules.

Supplementary Figure 11 Boxplot of the distribution of 24 immune cells among three groups in the METABRIC cohort (N.S. represents no significance, **p* < 0.05, ***p* < 0.01, ****p* < 0.001, and *****p* < 0.0001).

Supplementary Figure 12 Boxplot of the distribution of 10 immune-related cells and five tumor progression-related pathways among three groups in the METABRIC cohort (N.S. represents no significance, **p* < 0.05, ***p* < 0.01, ****p* < 0.001, and *****p* < 0.0001).

Supplementary Figure 13 Characteristics of identified subclasses in the METABRIC cohort. **(A)** Heatmap of the top 10 marker genes of each subclass in the METABRIC cohort. **(B)** Bar plot of variant classification distributions among METABRIC ER+ BC patients. **(C)** Bar plot of variant type distributions among METABRIC ER+ BC patients. **(D)** Oncoprint of mutation status of top 30 highly mutated genes across three subtypes (left) and ordered by mutation load (right). **(E)** Kaplan–Meier curves of three identified subclasses.

Supplementary Figure 14 Boxplot of the distribution of 34 checkpoint molecules among three groups in the METABRIC cohort (N.S. represents no significance, **p* < 0.05, ***p* < 0.01, ****p* < 0.001, and *****p* < 0.0001).

Supplementary Figure 15 Coloring single samples on a dimensional reduction plot according to 23 checkpoint molecules in the METABRIC cohort. **(A)** The t-SNE distribution of METABRIC ER+ BC samples by expression profile of global immune genes. Each point represents a single sample; different colors represent the three subclasses. **(B–X)** Coloring single samples on a dimensional reduction plot according to 23 checkpoint molecules.

Supplementary Table 1 Clinical information of enrolled patients.

Supplementary Table 2 Identification of driver genes based on the TCGA ER+ mutation data.

Supplementary Table 3 Identification of the marker genes of each subclass in the TCGA cohort.

Supplementary Table 4 Datasets for SubMap analysis. **(A)** Melanoma dataset. **(B)** GDSC dataset. **(C)** TCGA dataset. **(D)** GEO dataset. **(E)** METABRIC dataset.

Supplementary Table 5 Single-cell annotation results by Azizi and Garvan.

## Abbreviations

BC: breast cancer
CNVs: copy number variations
SNP: single-nucleotide polymorphism
TCGA: The Cancer Genome Atlas
TMB: tumor mutation burden
TILs: tumor-infiltrating lymphocytes
PFS: progression-free survival
DFS: disease-free survival
DLDA: diagonal linear discriminant analysis
TGF-β: transforming growth factor-β
EMT: epithelial–mesenchymal transition.
