# Supplementary figures and images for: Classification of Estrogen Receptor-Positive Breast Cancer Based on Immunogenomic Profiling and Validation at Single-Cell Resolution
Source: Front Cell Dev Biol. 2021 Sep 21;9:722841. doi: 10.3389/fcell.2021.722841 (PMC8490889; doi:10.3389/fcell.2021.722841)

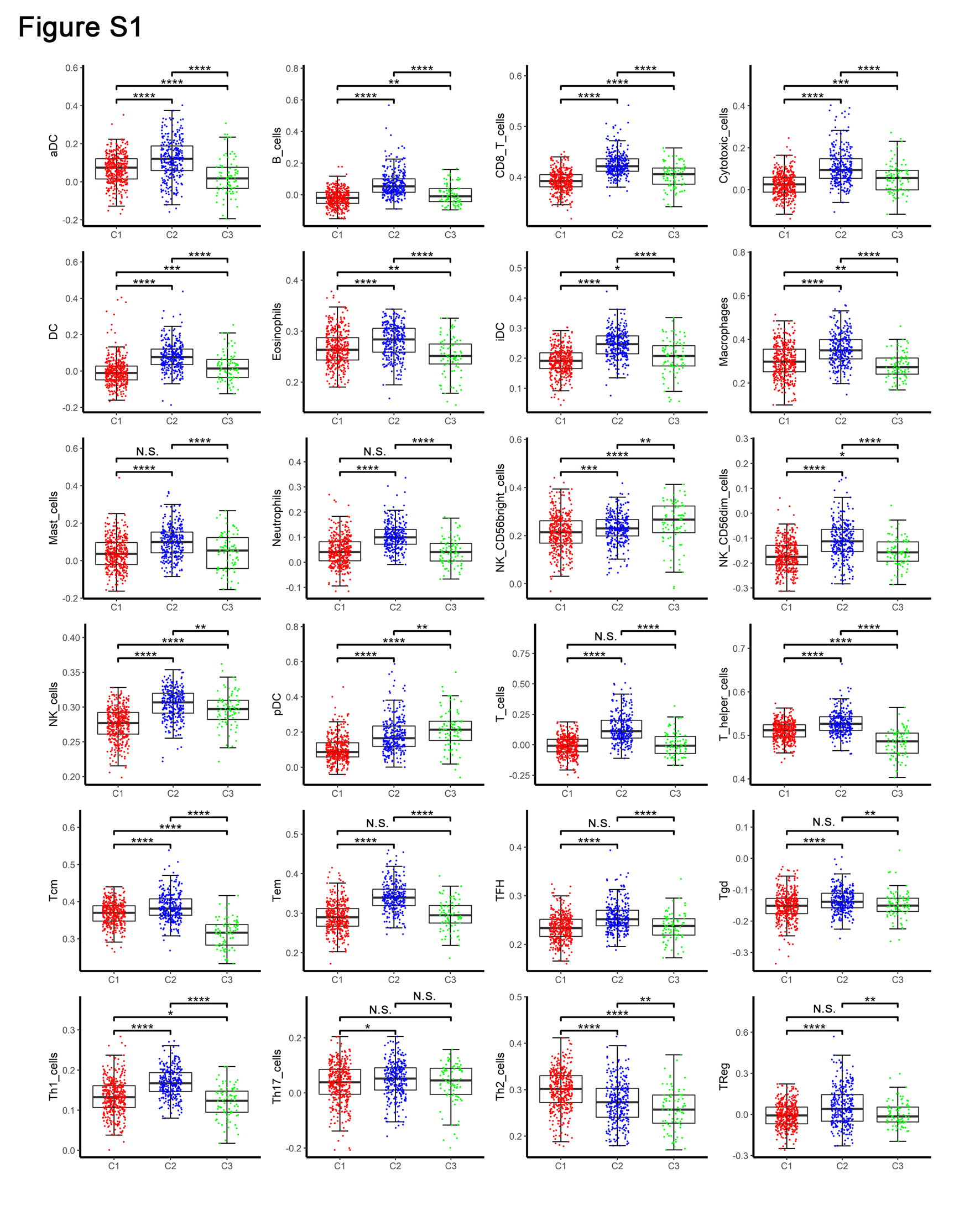

Supplement: Supplementary Figure 1 — Boxplot of the distribution of 24 immune cells among three groups in the TCGA cohort (N.S. represents no significance, *p < 0.05, **p < 0.01, ***p < 0.001, and ****p < 0.0001). [file Image_1.TIF]

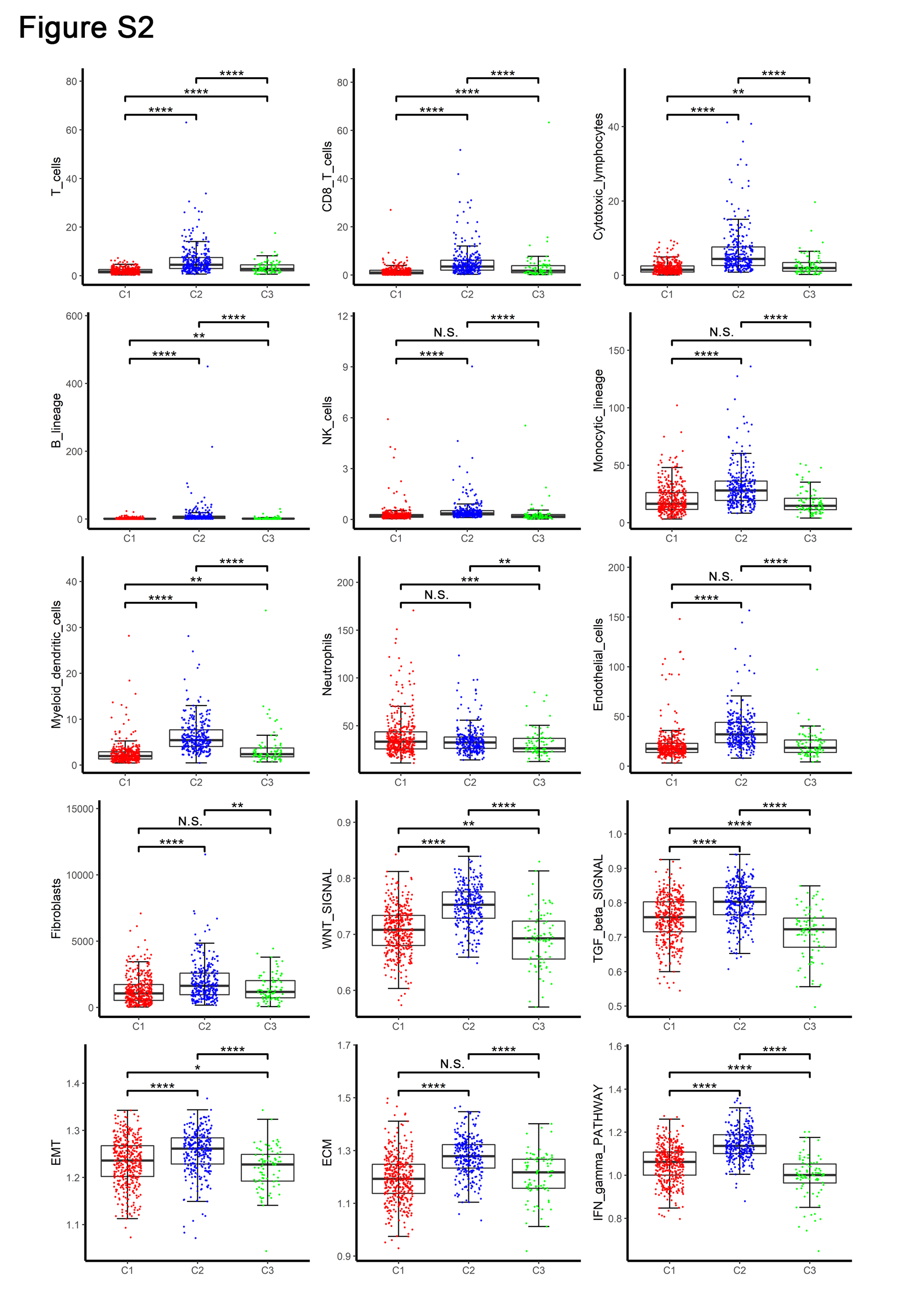

Supplement: Supplementary Figure 2 — Boxplot of the distribution of 10 immune-related cells and five tumor progression-related pathways among three groups in the TCGA cohort (N.S. represents no significance, *p < 0.05, **p < 0.01, ***p < 0.001, and ****p < 0.0001). [file Image_2.TIF]

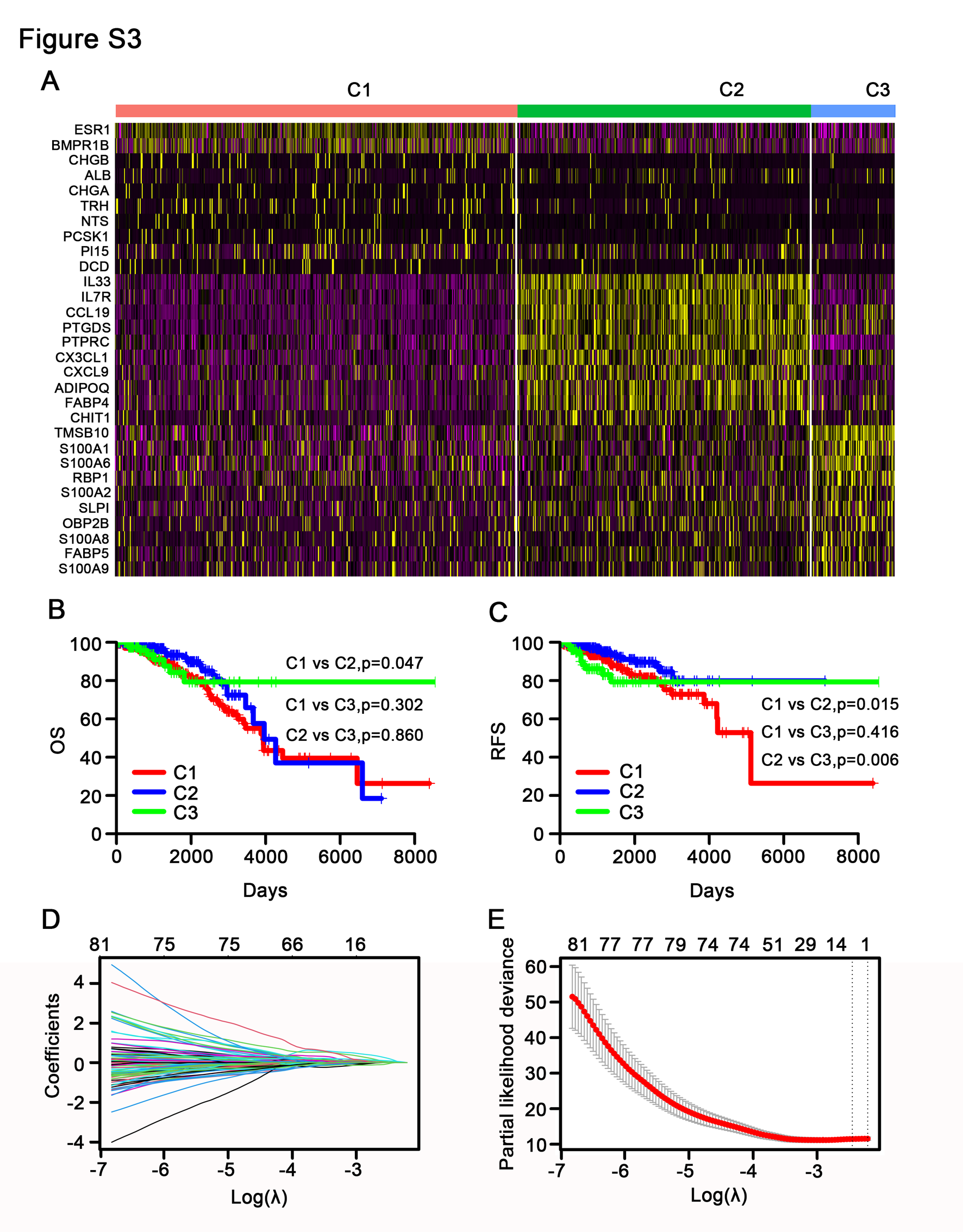

Supplement: Supplementary Figure 3 — Heatmap of the top 10 marker genes of each subclass in the TCGA cohort (A) and Kaplan–Meier curves of each subclass (B,C). Candidate immune gene selection using the LASSO regression model (D,E). [file Image_3.TIF]

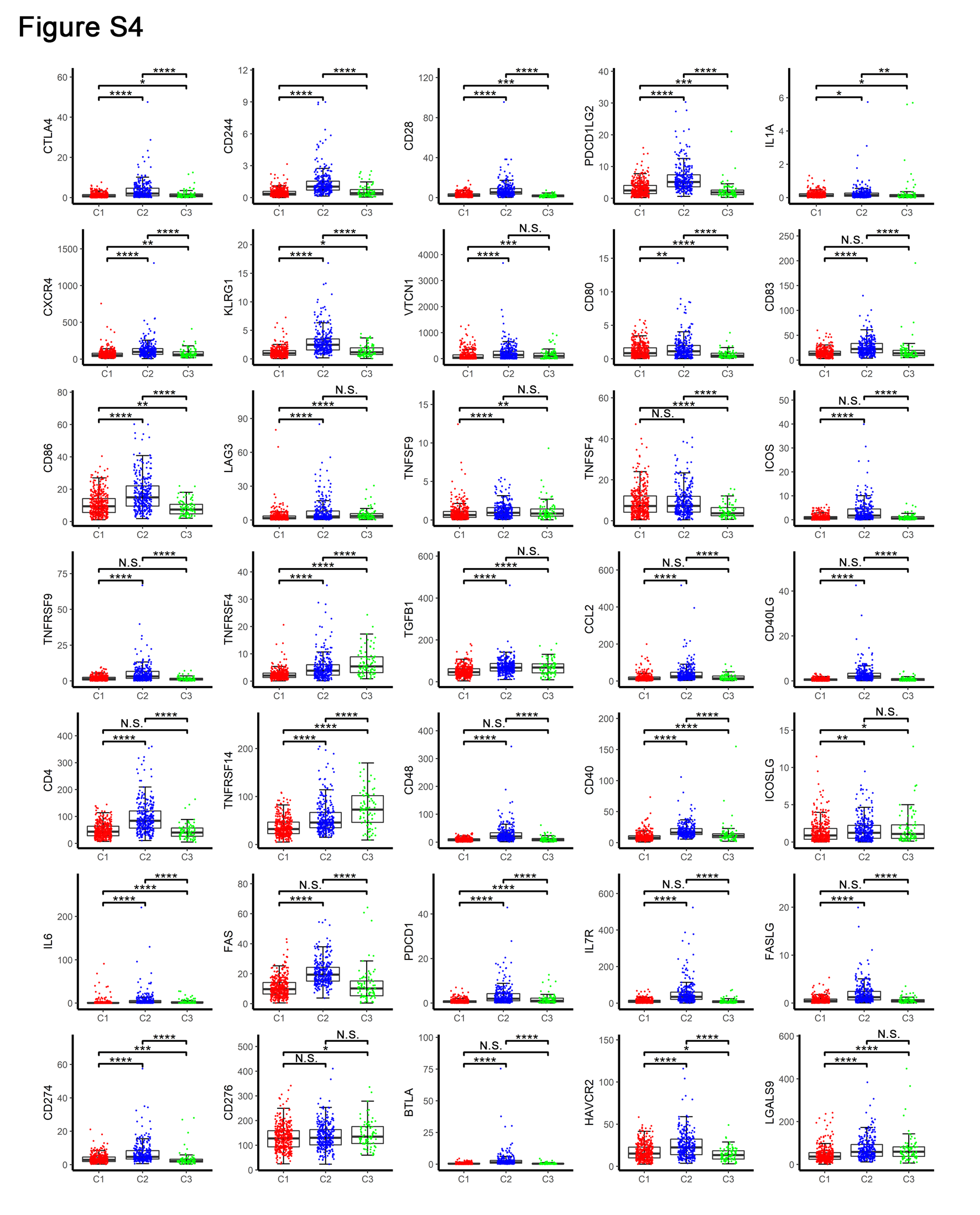

Supplement: Supplementary Figure 4 — Boxplot of the distribution of 35 checkpoint molecules among three groups in the TCGA cohort (N.S. represents no significance, *p < 0.05, **p < 0.01, ***p < 0.001, and ****p < 0.0001). [file Image_4.TIF]

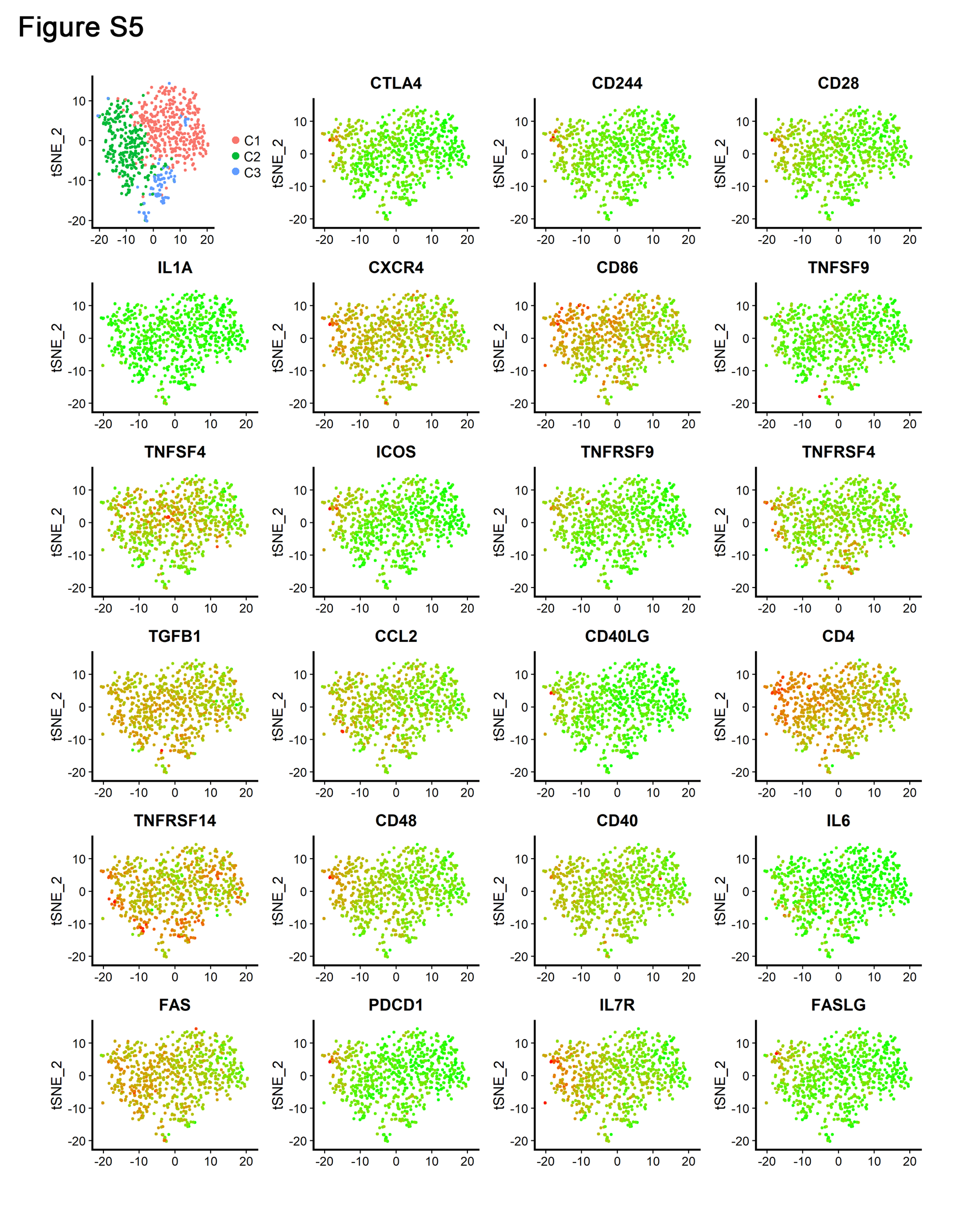

Supplement: Supplementary Figure 5 — Coloring single samples on a dimensional reduction plot according to 23 checkpoint molecules in the TCGA cohort. (A) The t-SNE distribution of TCGA ER+ BC samples by expression profile of global immune genes. Each point represents a single sample; different colors represent the three subclasses. (B–X) Coloring single samples on a dimensional reduction plot according to 23 checkpoint molecules. [file Image_5.TIF]

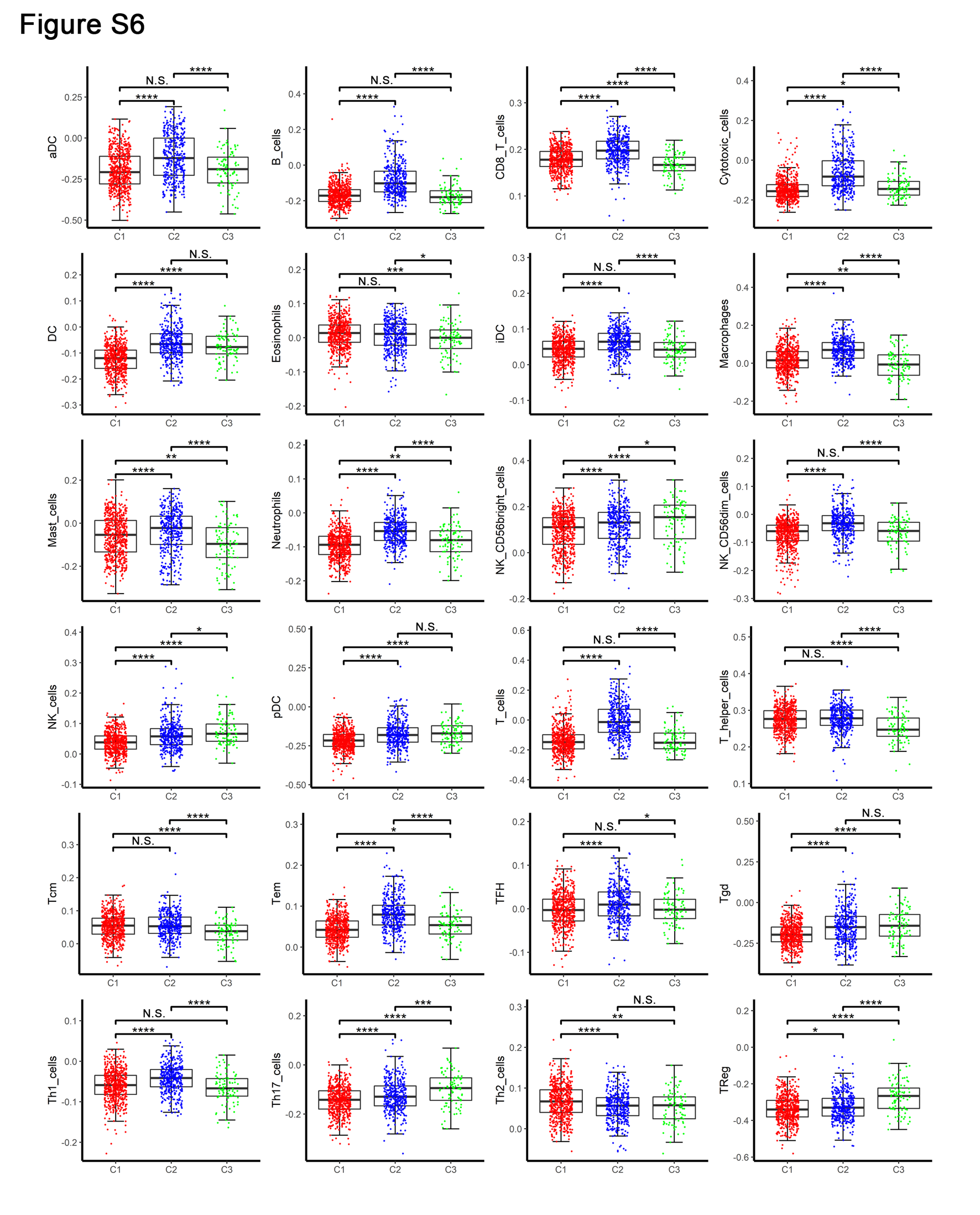

Supplement: Supplementary Figure 6 — Boxplot of the distribution of 24 immune cells among three groups in the GEO-meta cohort (N.S. represents no significance, *p < 0.05, **p < 0.01, ***p < 0.001, and ****p < 0.0001). [file Image_6.TIF]

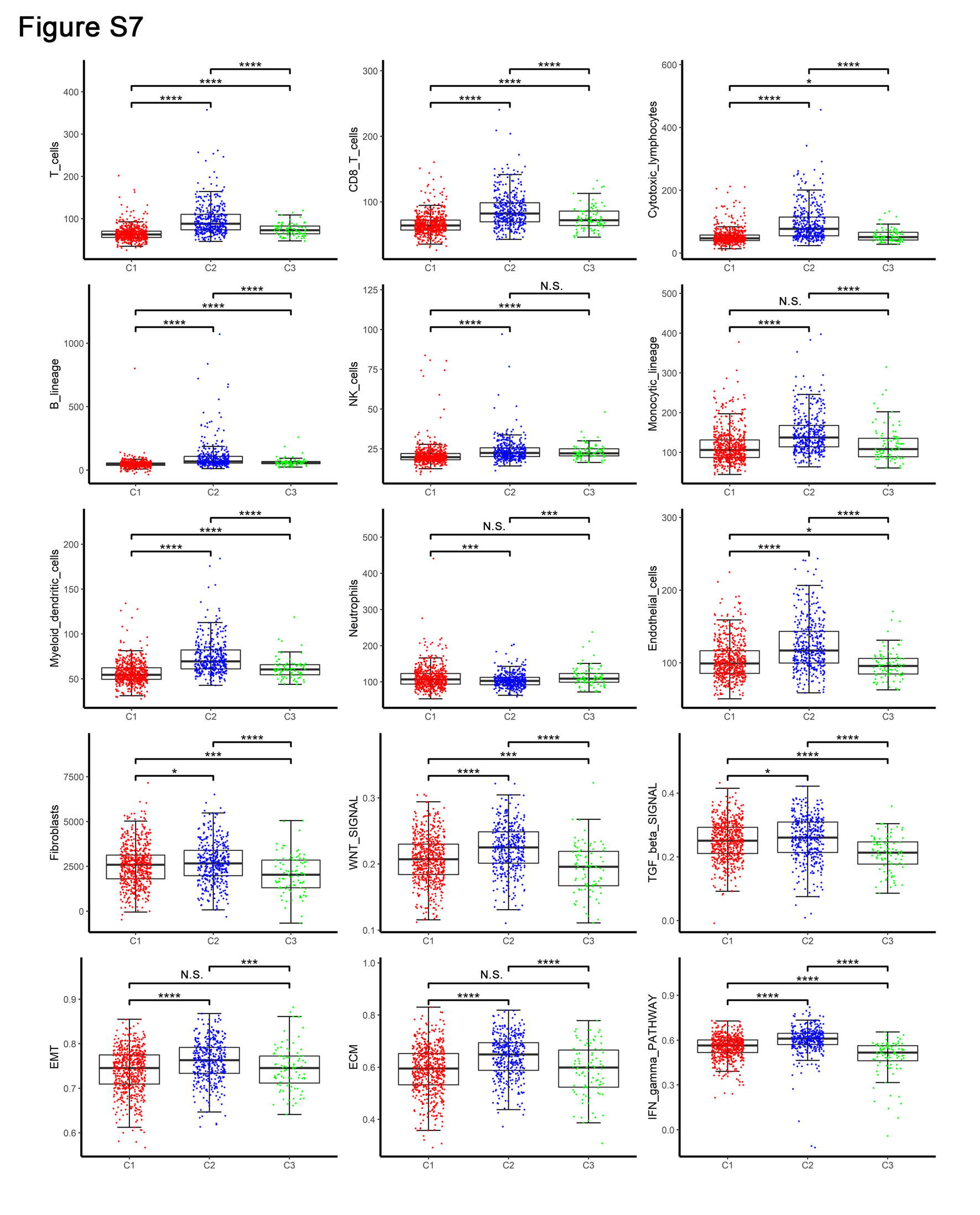

Supplement: Supplementary Figure 7 — Boxplot of the distribution of 10 immune-related cells and five tumor progression-related pathways among three groups in the GEO-meta cohort (N.S. represents no significance, *p < 0.05, **p < 0.01, ***p < 0.001, and ****p < 0.0001). [file Image_7.TIF]

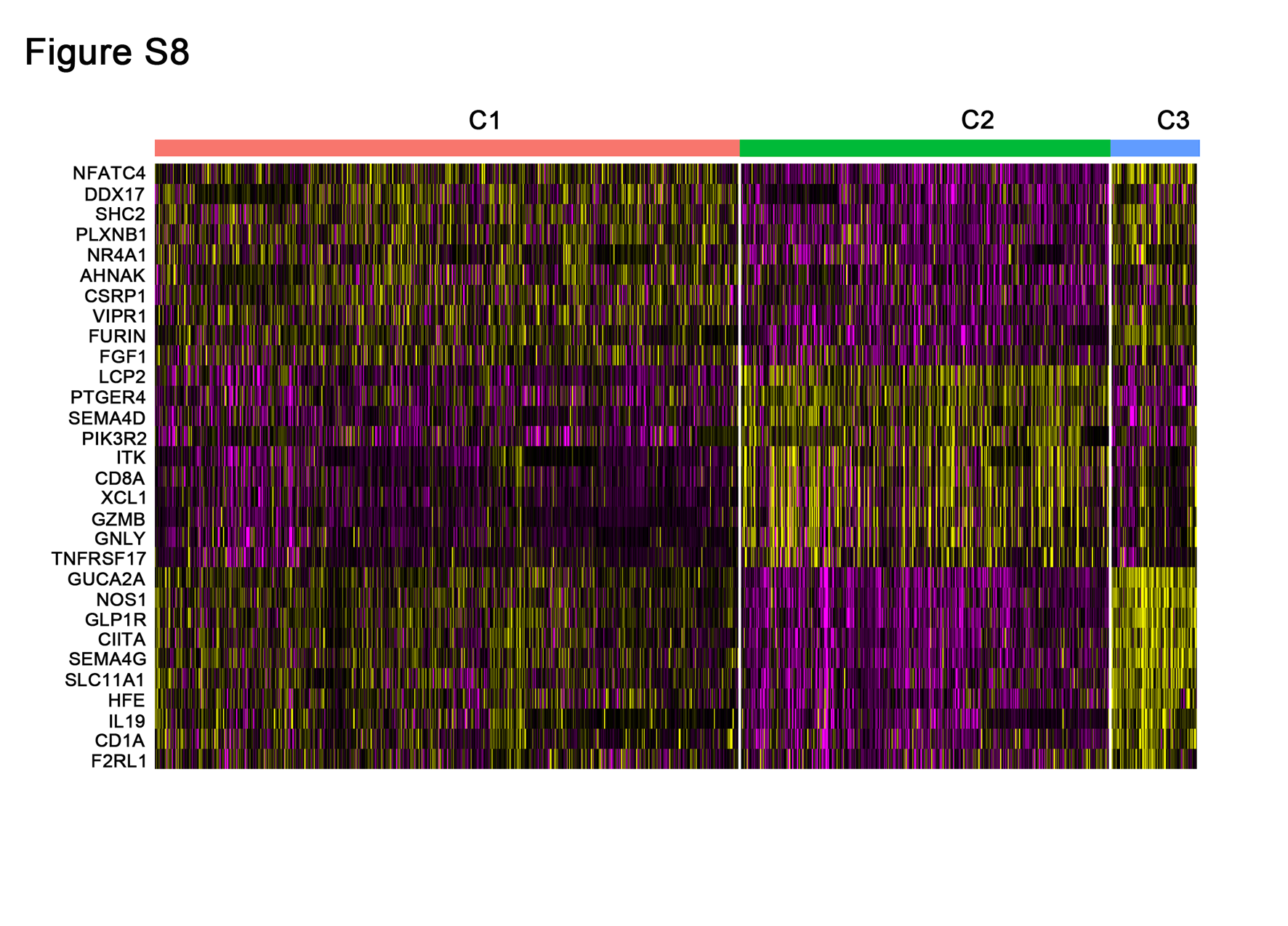

Supplement: Supplementary Figure 8 — Heatmap of the top 10 marker genes of each subclass in the GEO-meta cohort (A) and Kaplan–Meier curves of each subclass (B). [file Image_8.TIF]

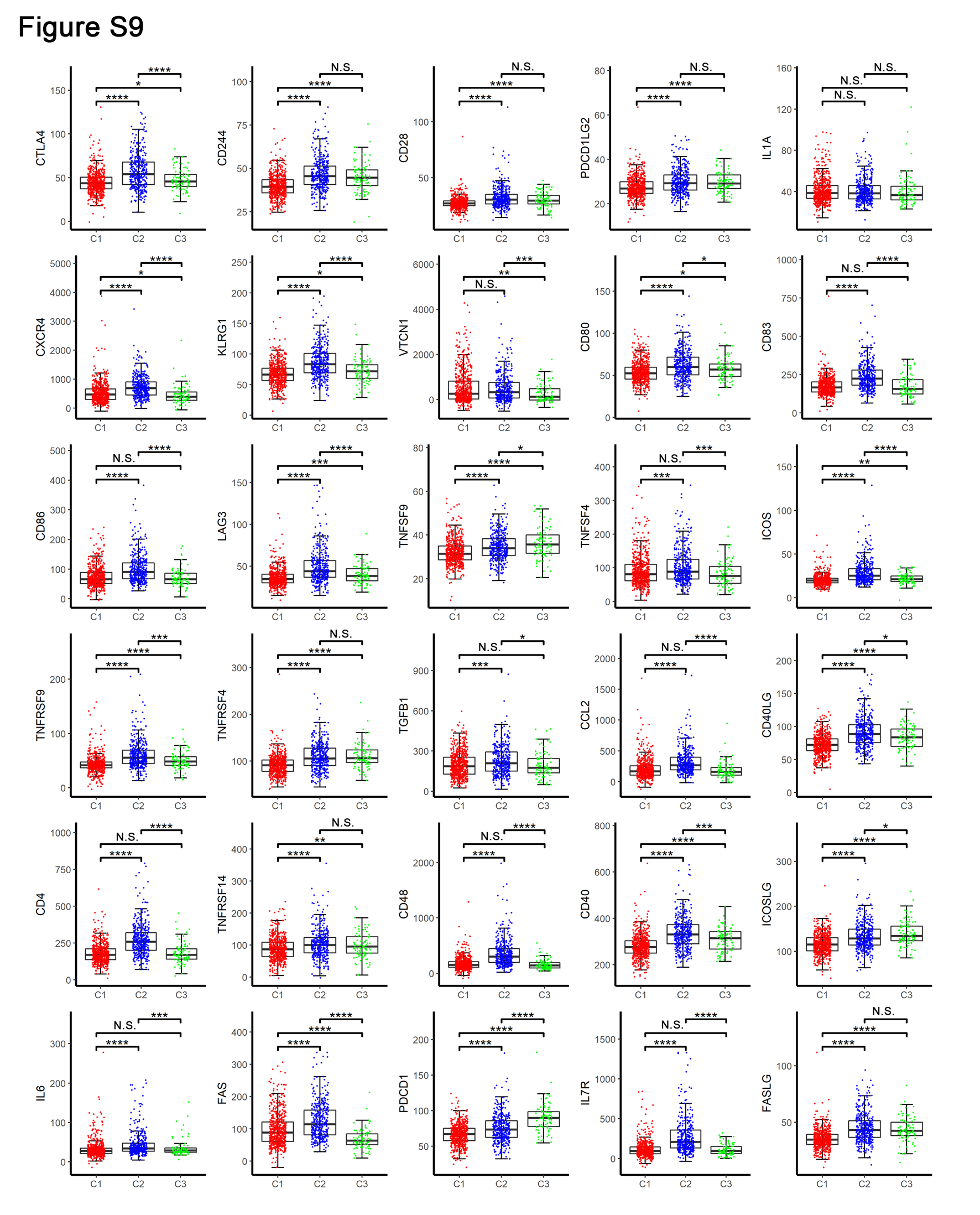

Supplement: Supplementary Figure 9 — Boxplot of the distribution of 30 checkpoint molecules among three groups in the GEO-meta cohort (N.S. represents no significance, *p < 0.05, **p < 0.01, ***p < 0.001, and ****p < 0.0001). [file Image_9.TIF]

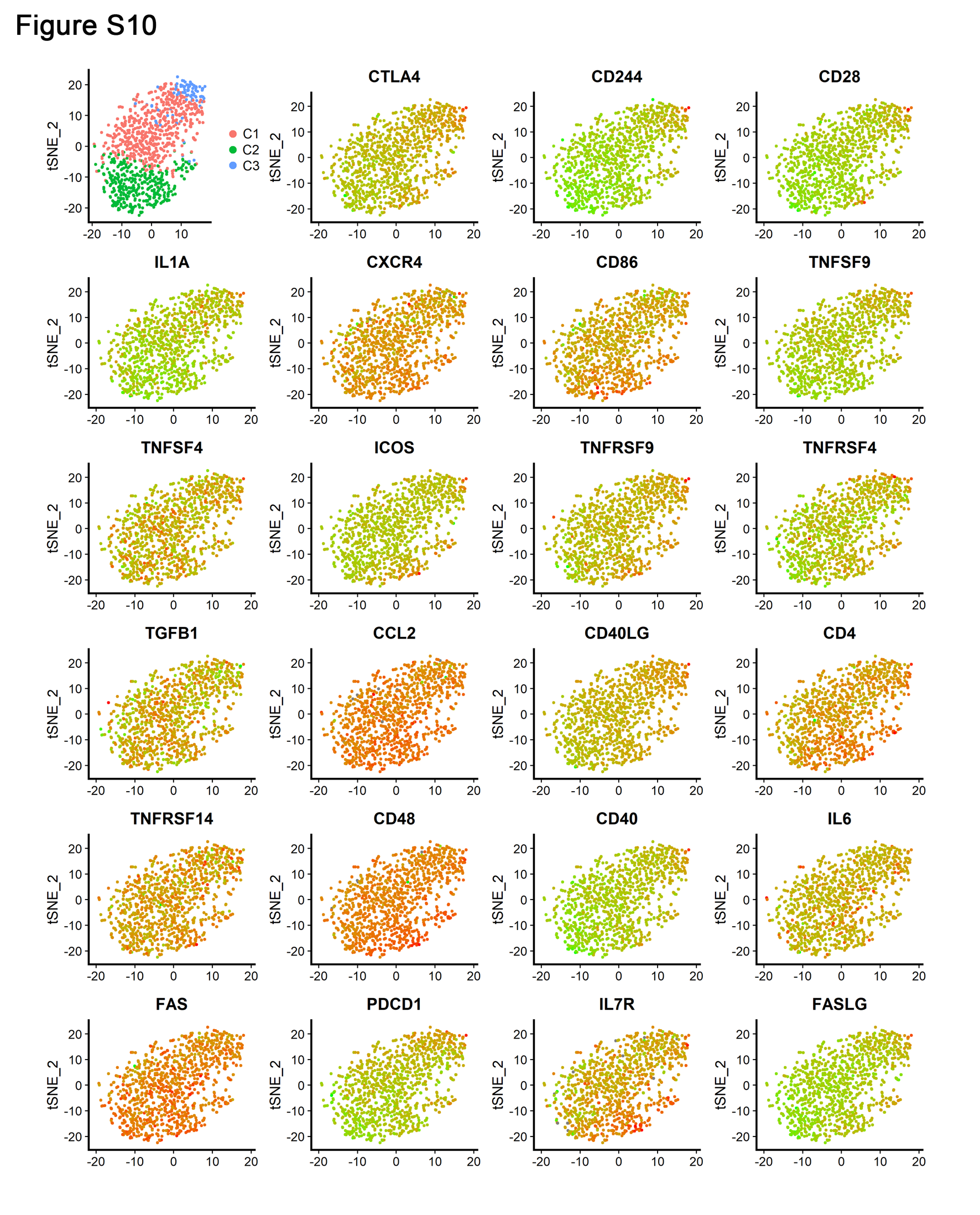

Supplement: Supplementary Figure 10 — Coloring single samples on a dimensional reduction plot according to 23 checkpoint molecules in the GEO-meta cohort. (A) The t-SNE distribution of GEO-meta ER+ BC samples by expression profile of global immune genes. Each point represents a single sample; different colors represent the three subclasses. (B–X) Coloring single samples on a dimensional reduction plot according to 23 checkpoint molecules. [file Image_10.TIF]

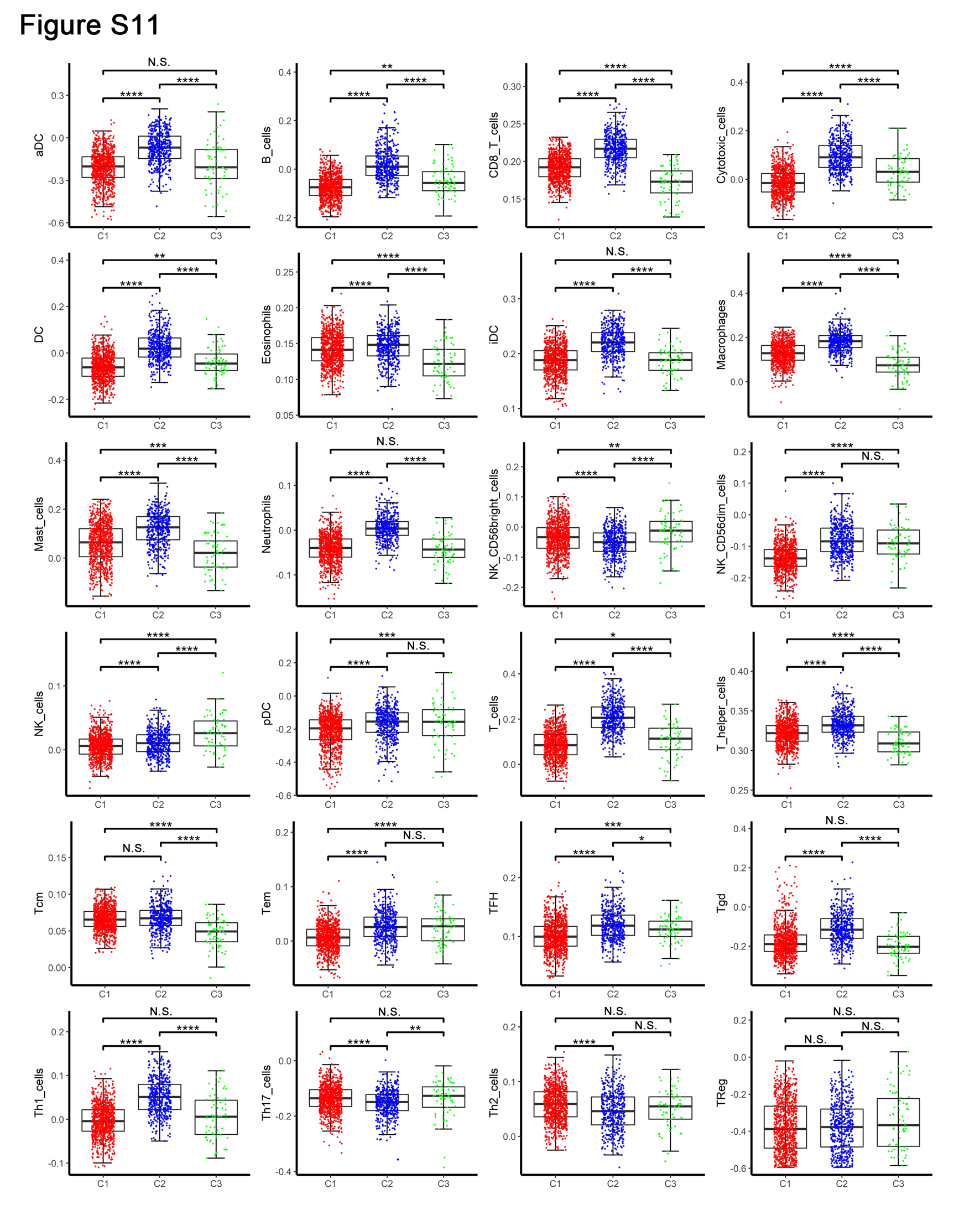

Supplement: Supplementary Figure 11 — Boxplot of the distribution of 24 immune cells among three groups in the METABRIC cohort (N.S. represents no significance, *p < 0.05, **p < 0.01, ***p < 0.001, and ****p < 0.0001). [file Image_11.TIF]

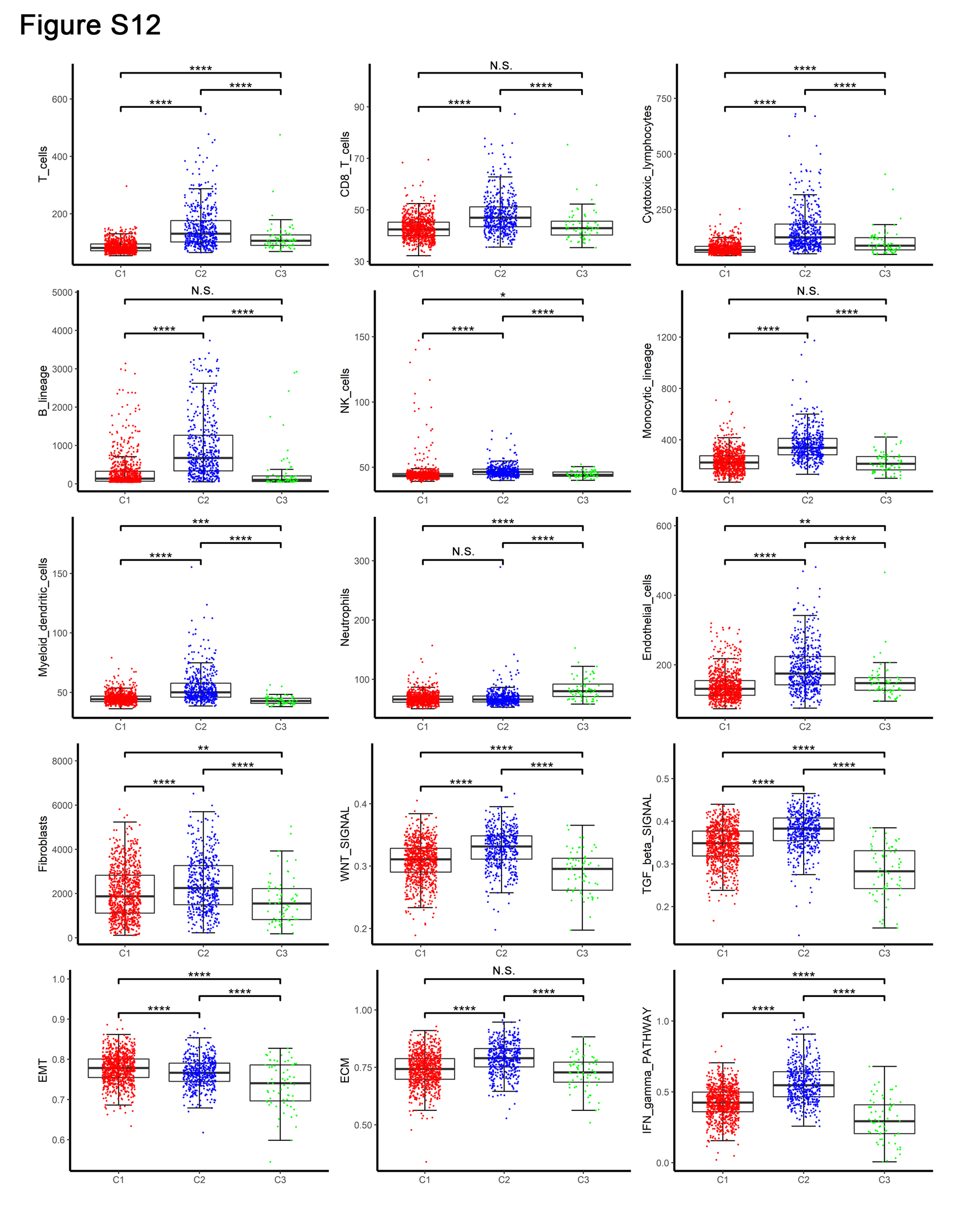

Supplement: Supplementary Figure 12 — Boxplot of the distribution of 10 immune-related cells and five tumor progression-related pathways among three groups in the METABRIC cohort (N.S. represents no significance, *p < 0.05, **p < 0.01, ***p < 0.001, and ****p < 0.0001). [file Image_12.TIF]

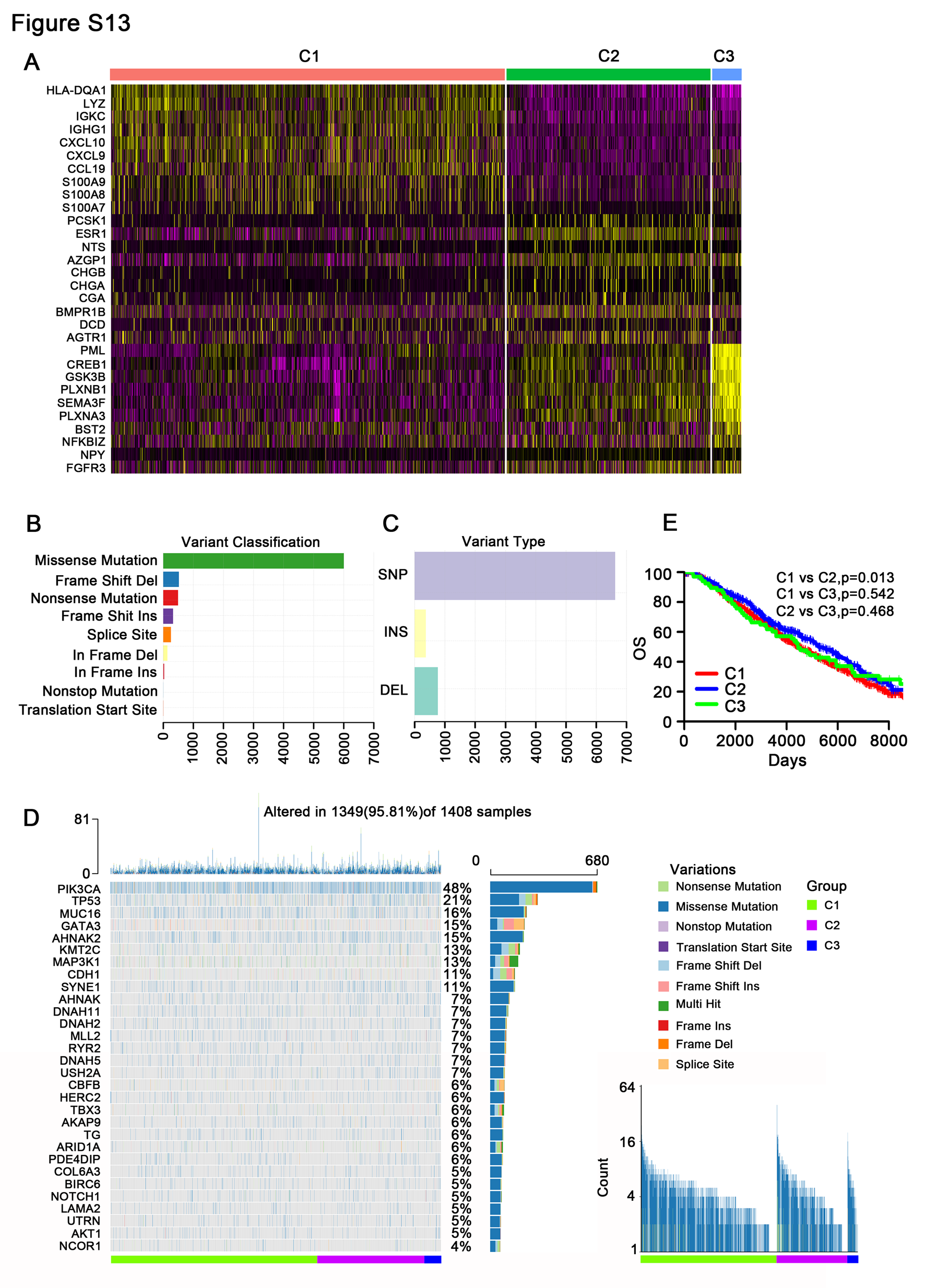

Supplement: Supplementary Figure 13 — Characteristics of identified subclasses in the METABRIC cohort. (A) Heatmap of the top 10 marker genes of each subclass in the METABRIC cohort. (B) Bar plot of variant classification distributions among METABRIC ER+ BC patients. (C) Bar plot of variant type distributions among METABRIC ER+ BC patients. (D) Oncoprint of mutation status of top 30 highly mutated genes across three subtypes (left) and ordered by mutation load (right). (E) Kaplan–Meier curves of three identified subclasses. [file Image_13.TIF]

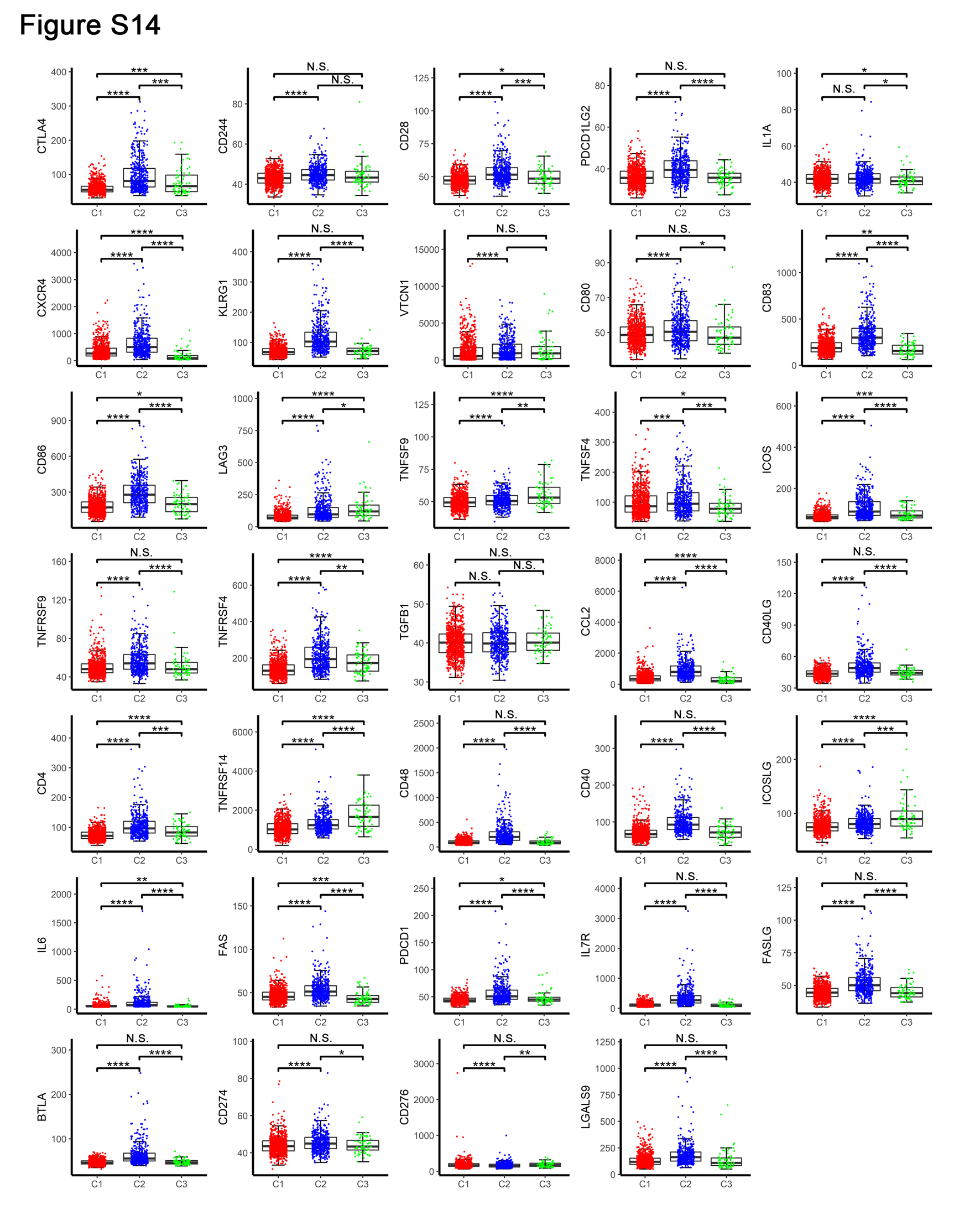

Supplement: Supplementary Figure 14 — Boxplot of the distribution of 34 checkpoint molecules among three groups in the METABRIC cohort (N.S. represents no significance, *p < 0.05, **p < 0.01, ***p < 0.001, and ****p < 0.0001). [file Image_14.TIF]

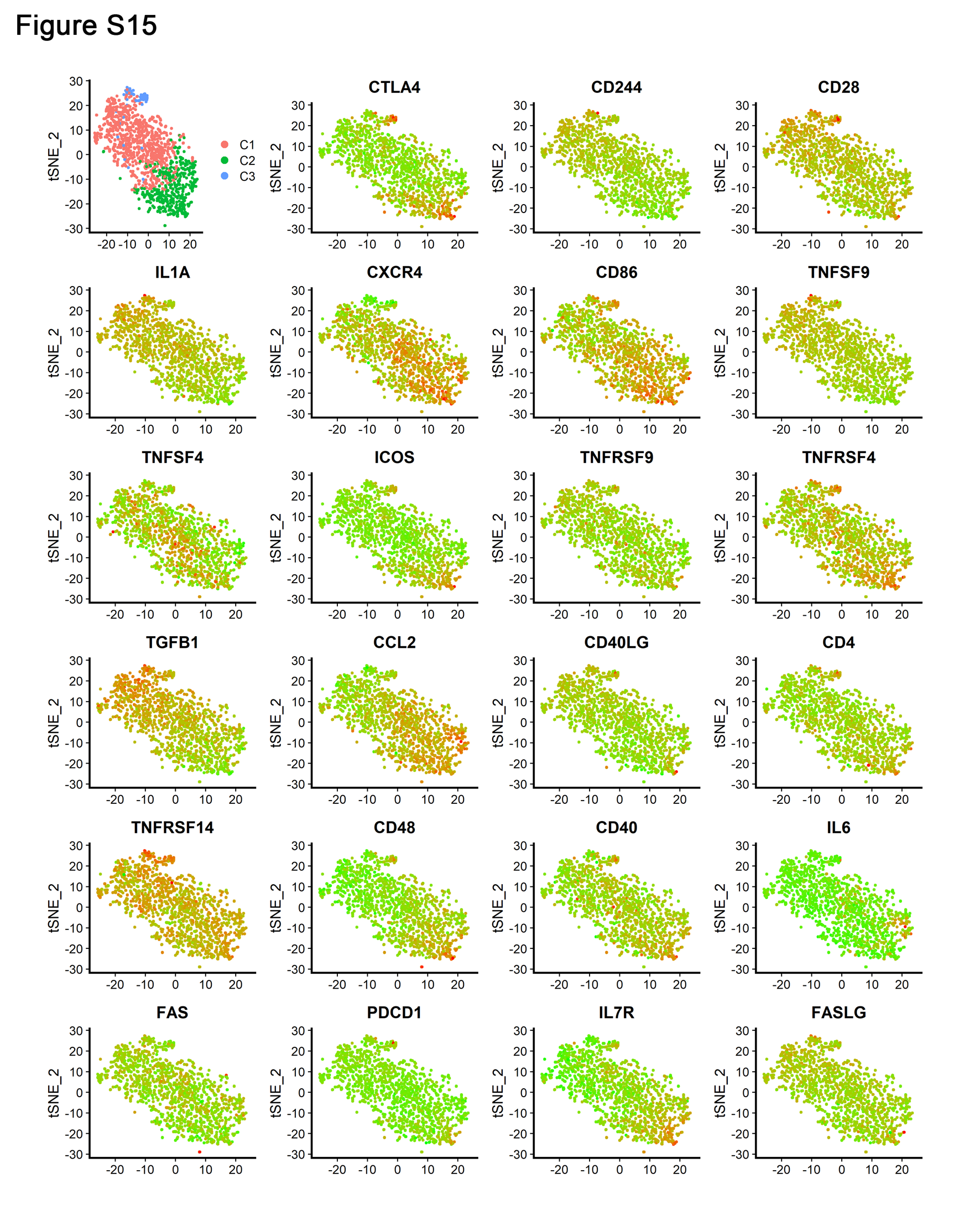

Supplement: Supplementary Figure 15 — Coloring single samples on a dimensional reduction plot according to 23 checkpoint molecules in the METABRIC cohort. (A) The t-SNE distribution of METABRIC ER+ BC samples by expression profile of global immune genes. Each point represents a single sample; different colors represent the three subclasses. (B–X) Coloring single samples on a dimensional reduction plot according to 23 checkpoint molecules. [file Image_15.TIF]
